# Microglial PGC-1α alleviates synaptic damage and cognitive impairments following anesthesia and surgery by suppressing excessive synaptic pruning in aged mice

**DOI:** 10.7150/ijbs.121472

**Published:** 2026-03-25

**Authors:** Xuyang Wu, Maokai Xu, Yongxin Huang, Pinzhong Chen, Liang Wu, Andi Chen, Jianjie Wei, Yingjie Chen, Yongbao Lin, Yingceng Lin, Zhe Lin, Ying Lin, Fushan Xue, Xiaohui Chen, Xiaochun Zheng

**Affiliations:** 1 Department of Anesthesiology, Shengli Clinical Medical College of Fujian Medical University, Fujian Provincial Hospital, Fuzhou University Affiliated Provincial Hospital, Fuzhou, China.; 2 Department of Anesthesiology, Fuzhou Second General Hospital, Fuzhou, China.; 3 School of Basic Medical Sciences, Fujian Medical University, Fuzhou, China.; 4 Fujian Emergency Medical Center, Fujian Provincial Key Laboratory of Emergency Medicine, Fujian Provincial Key Laboratory of Critical Care Medicine, Fujian Provincial Co-Constructed Laboratory of “Belt and Road”, Fuzhou, China.

**Keywords:** Postoperative cognitive dysfunction, peroxisome proliferators-activated receptor γ coactivator-1α, Microglia, Synaptic pruning, Mitochondrial energy metabolism

## Abstract

Postoperative cognitive dysfunction (POCD) in the elderly is a serious clinical concern. Although microglial phagocytosis is known to depend on mitochondrial metabolism, and its dysregulation can lead to abnormal synaptic pruning and neuronal injury, the molecular link between these processes in POCD pathogenesis requires further elucidation. In this study, we established a POCD animal model of aged mice using isoflurane exposure and partial hepatectomy to investigate how anesthesia and surgery impacted synaptic plasticity via microglial phagocytosis. Our findings demonstrated that anesthesia and surgery significantly reduced hippocampal peroxisome proliferators-activated receptor γ coactivator-1α (PGC-1α) expression, leading to impaired mitochondrial energy metabolism, abnormal microglial phagocytosis and excessive synaptic pruning, which was associated with synaptic deficits and cognitive dysfunction. Importantly, the treatment with the PGC-1α activator ZLN005 or AAV-mediated overexpression of PGC-1α not only successfully restored PGC-1α level in the hippocampus of aged mice, but also effectively ameliorated mitochondrial dysfunction, reversed abnormal microglia-mediated synaptic pruning, restored synaptic plasticity, and improved POCD. Our findings identify microglial PGC-1α as a critical mediator in the pathogenesis of POCD, linking mitochondrial energy metabolism with microglia-mediated synaptic pruning, and highlight the potential of microglial PGC-1α as a promising therapeutic target for prevention and treatment of POCD.

## Introduction

Postoperative cognitive dysfunction (POCD) is a central nervous system complication frequently observed in elderly patients after anesthesia and surgery. It is mainly characterized by symptoms including anxiety, cognitive impairments, memory impairments, and psychological dysfunction 1. This condition may last for days to months after surgery, and even some patients may develop long-term cognitive deficits, resulting in delayed postoperative recovery and prolonged hospitalization. Recent advancements in anesthesia techniques and perioperative management have resulted in continuous improvement of patient outcome 2, 3. However, the pathogenesis of POCD remains incompletely understood, and there is still no consensus about the specific biomarkers for clinical diagnosis of POCD. The current assessments predominantly rely on neuropsychological scales, such as the Mini-Mental State Examination and the Montreal Cognitive Assessment, but their sensitivity and standardization are subjects of ongoing debate 4, 5. The absence of compelling molecular targets for the early diagnosis of POCD further complicates its prevention and treatment, thereby making it a significant focus for researchers in perioperative care.

The current academic consensus holds that the occurrence and progression of POCD in elderly patients are the result of the combined effects of external factors such as anesthesia, surgery, and related complications, based on natural degenerative changes in the central nervous system 6. Both anesthesia and surgery may cause memory impairments by directly or indirectly influencing neuronal activity, and previous studies have primarily focused on the direct effects of anesthesia and surgery on neuronal excitability using surgical mouse models 7. However, deficits in synaptic plasticity, especially reduced dendritic-spine density in hippocampal neurons and suppressed long-term potentiation, appears to contribute substantially to the onset and progression of POCD 8, 9. Emerging evidence challenges the neuron-centric view by highlighting neuroimmune crosstalk: activated microglia have been implicated in structural synaptic loss 10. Indeed, in recent POCD models, hippocampal synaptic loss has been correlated with microglial activation rather than neuronal apoptosis, indicating that microglia-mediated synaptic damage plays a central role in postoperative cognitive decline 11.

Extensive researches have shown that microglia regulate neuronal growth, survival, and synaptic remodeling in the central nervous system through various mechanisms, including release of inflammatory mediators and activation of complement system 12, 13. Furthermore, microglia can also modulate neuronal activity by reshaping synaptic structures 14. Growing evidence indicates that microglia sense neuronal activity and actively phagocytose weak or immature synapses ina process known as synaptic pruning 15, 16. Synaptic pruning is a natural mechanism vital for neural circuit refinement during development. However, excessive pruning in the adult brain can be harmful and has been linked to synapse loss in several pathological conditions 17, 18.

Mitochondrial energy metabolism is essential for normal microglial phagocytic activity, as oxidative phosphorylation (OXPHOS) provides the ATP required for energy-intensive processes such as cytoskeletal remodeling and engulfment. Although adequate OXPHOS and ATP levels generally support microglial functions including phagocytosis, impaired mitochondrial metabolism does not always suppress microglial activity; under certain stress conditions, it can instead drive a reactive phenotype with increased engulfment 19, 20. In the states of metabolic stress resulting from impaired oxidative phosphorylation and decreased ATP levels, disrupted electron transport can increase the production of mitochondrial reactive oxygen species (ROS), which in turn activate stress-responsive signaling pathways and pro-inflammatory transcriptional programs. Impaired mitochondrial function has been linked to the activation of MAPK and NF-κB signaling cascades, promoting pro-inflammatory cytokine expression and microglial activation 21, 22. Elevated ROS and downstream inflammatory signaling can shift microglia toward a hyperactivated, pro-inflammatory state that exhibits enhanced synaptic or neuronal engulfment. Such mechanisms have been observed in multiple models of neurodegeneration and brain injury, where microglial mitochondrial dysfunction is linked not only to dysregulated immune activation and ROS production but also to excessive or aberrant phagocytic behavior 23, 24. These findings underscore the notions that regulation of energy metabolism is a critical determinant of microglial phagocytic function and may contribute to pathological processes such as abnormal synaptic pruning and neuroinflammation in disease states.

One key regulator of mitochondrial function is peroxisome proliferator-activated receptor γ coactivator-1α (PGC-1α), a transcriptional coactivator that plays a central role in stimulating mitochondrial biogenesis and respiration 25, 26. By up-regulating nuclear respiratory factors (NRFs) and mitochondrial transcription factor A (TFAM), PGC-1α drives mitochondrial regeneration and enhances cellular energy metabolism 27. In the central nervous system, PGC-1α is expressed predominantly in neurons, microglia, and astrocytes, especially in the regions such as cerebral cortex and hippocampus, and its expression in microglia can change dynamically in response to microenvironmental alterations such as neuroinflammatory signals or metabolic stress 28. Other than the well-established role in energy homeostasis, PGC-1α has recently been demonstrated as a potent suppressor of inflammation and oxidative stress 29, 30. For example, activation of PGC-1α can polarize the microglia toward an anti-inflammatory phenotype, enhance antioxidant defenses, and promote mitochondrial turnover through mitophagy and autophagy, thereby restraining inflammasome activation and reducing pro-inflammatory cytokine production 31. In the neurodegenerative or neural injury models, PGC-1α activation has also been shown to ameliorate mitochondrial dysfunction and reduce neurotoxic insults, indicating its therapeutic potential 32, 33. Given the crucial role of microglia in synaptic pruning and their dependence on mitochondrial energy metabolism, enhancing PGC-1α to optimize microglial metabolism and phenotype may represent a promising strategy for prevention or treatment of POCD. Nonetheless, despite accumulating evidence on the interlinkage between mitochondrial health and cognitive function, the specific role of PGC-1α in regulating microglial activity in the context of POCD remains underexplored.

This study was designed to determine the potential of PGC-1α as a therapeutic target to enhance mitochondrial energy metabolism in microglia, thereby preventing excessive synaptic pruning and preserving cognitive function postoperatively. By exploring the molecular mechanisms through which PGC-1α regulates synaptic pruning in microglia, we aim to uncover its causal role in the pathogenesis of POCD and provide a promising approach for maintaining perioperative cognitive health in elderly patients.

## Methods

### Animals

Aged C57BL/6J male mice (18-20 months old) were obtained from the Fujian Provincial Laboratory Animal Center. They were kept in a controlled environment with a 12-hour light-dark cycle, with lights on at 6:00 AM, and maintained at a constant temperature of 22-24°C and humidity of 50-60%. Before any experimental procedure, the animals were allowed to acclimate to the housing conditions for about 2 weeks, with five mice per cage and free access to food and water. In all experiments, age-matched male littermates were used, following the same treatment protocols. Experimenters were blinded to the group assignments to ensure objectivity and avoid bias. All protocols received approval from the Animal Ethics Committee of the Fujian Provincial Laboratory Animal Center (IACUC FPH-SL-20231020[0157]) and adhered to the NIH guidelines for the Care and Use of Laboratory Animals. The design of the experiment was adjusted to decrease the number of animals and to reduce their suffering, thereby ensuring adherence to ethical standards.

### POCD paradigm

Following previously reported methods 34, 35, a POCD model of aged mice was established by partial hepatectomy under 1.5% isoflurane anesthesia. After adequate anesthesia was achieved by monitoring with an anesthesia machine, a midline abdominal incision was made to expose the abdominal organs. The left lateral lobe of the liver, accounting for 30% of the total liver mass, was ligated and resected. Subsequently, the abdominal organs were replaced into the cavity, and the muscle layer and skin were sutured using 3-0 sutures. The incision was disinfected and covered with mupirocin gauze. Anesthesia was maintained until the completion of the procedure. Additional anesthesia was allowed to alleviate pain, if necessary. A standardized surgeon performed the operation to reduce variability. The surgical procedure lasted approximately 20 minutes, with a total anesthesia time of 2.5 hours. Postoperatively, mouse was housed in individually ventilated cage (IVC) with *ad libitum* access to food and water. This model has been well validated, as it effectively simulates the postoperative state of aged mice who received anesthesia and surgery.

### Experimental protocol

**Figure 1:**
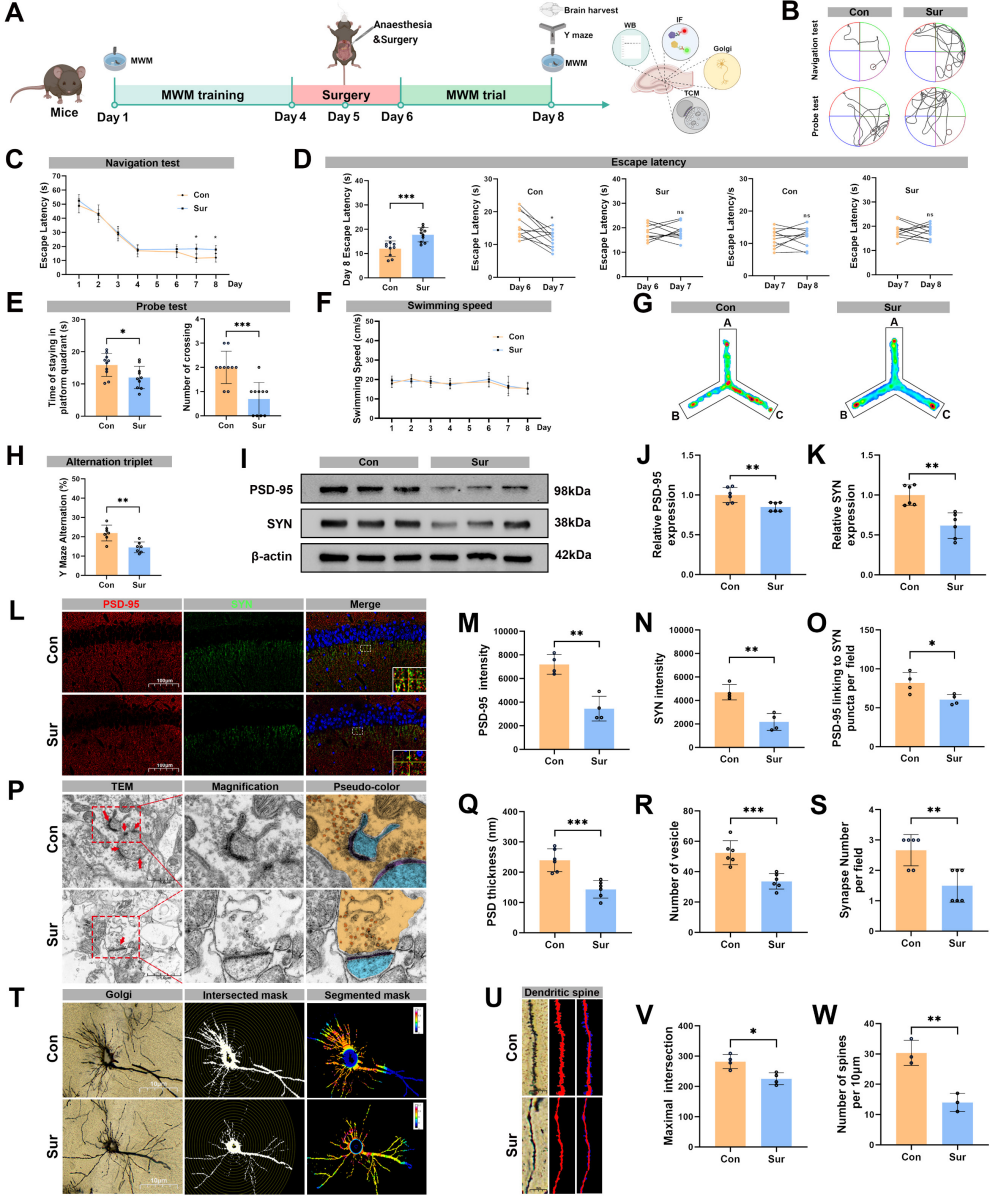
** Anesthesia and surgery induced cognitive dysfunction and impaired synaptic plasticity in aged mice. (**A) Experimental flowchart; (B) Trajectories of swimming in the MWM test; (C-D) Escape latency in the MWM test, n=10; (E) Time of staying on the platform and number of crossing the platform in the MWM test, n=10; (F) Swimming speed in the MWM test, n=10; (G) Heatmap of paths in the Y-maze test; (H) Y-maze spontaneous alternation rate, n=7; (I) Representative Western blots of PSD-95 and SYN in the hippocampus; (J-K) The relative expression levels of PSD-95 and SYN in the hippocampus, n=6; (L) Representative immunofluorescence images of synaptic PSD-95 and SYN in the hippocampal CA1 region; (M-N) Fluorescence intensity of PSD-95 and SYN in the hippocampal CA1 region, n=4; (O) Connection of PSD-95 and SYN puncta in each microscopic field of view, n=4; (P) Representative TEM images of synapses in the hippocampal CA1 region; (R-S) Quantificative analysis for the thickness of postsynaptic density, synaptic vesicles, and synapse number in the hippocampal CA1 region by TEM, n=6; (T) Representative Golgi-cox staining images of neurons in the hippocampal CA1 region; (U) Representative images for Golgi-cox staining with Sholl analysis of neurons in the hippocampal CA1 region; (V) Quantificative analysis for maximum number of intersection points in the hippocampal CA1 region, n=4; (W) Quantificative analysis for the number of dendritic spines, n=4; Data are presented as the mean ± SD. Student's t-test was used to obtain *p*-value. *p <0.05, **p < 0.01, ***p < 0.001, ns, not significant.

The study included three experimental sets. In the experiment 1, to investigate the effects of anesthesia and surgery on the synaptic plasticity and cognitive function in aged mice, as well as to observe the changes in the expression of PGC-1α and mitochondrial function, the mice were randomly assigned using a computer-generated randomization protocol into either a control (Con) or surgery (Sur) group. On the third day after surgery, hippocampal tissues were rapidly dissected, snap-frozen in liquid nitrogen, and stored at -80 °C for the single-nucleus RNA sequencing (snRNA-seq) and the bioinformatic analysis. For histological studies, tissues were fixed in 4% paraformaldehyde for 24 hours before embedding. The workflow of experiment 1 is shown in Figure 1A.

In the experiment 2, ZLN005 (MedChemExpress, New Jersey, USA), a selective and potent small-molecule activator of PGC-1α, was employed to determine whether pharmacological upregulation of PGC-1α could mitigate POCD in aged mice. Notably, ZLN005 is a brain-penetrant PGC-1α agonist with the ability of crossing the blood-brain barrier, thereby enabling direct modulation of PGC-1α signaling within the central nervous system 36. The mice were randomly assigned into three groups using a computer-generated randomization protocol: control with vehicle (ConVeh) group, surgery with vehicle (SurVeh) group, and surgery with ZLN005 (SurZLN) group. After behavioral training, the SurZLN group received an intraperitoneal injection of ZLN005 (1.5 mg/kg) 1 h prior to surgery, whereas the ConVeh and SurVeh groups were administered an equal volume of vehicle (PEG300; MedChemExpress) and saline, respectively. Subsequently, the SurVeh and SurZLN groups underwent anesthesia and surgery. Behavioral assessments were performed following surgery. On postoperative day 3, hippocampal tissues were harvested for molecular and histological analyses. The experimental timeline is illustrated in Figure 5A.

In the experiment 3, to specifically determine the causal role of microglia-specific PGC-1α upregulation in mediating mitochondrial energy metabolism and synaptic pruning in the hippocampus, an adeno-associated virus (AAV) carrying Ppargc1a under the control of the microglia-specific CX3CR1 promoter was employed to selectively enhance PGC-1α expression in the hippocampal microglia. The parameters associated with microglial mitochondrial energy metabolism and synaptic pruning were subsequently assessed. Using a computer-generated randomization protocol, mice were randomly assigned into three groups: control + AAV9-NC (ConAAV-NC) group, surgery + AAV9-NC (SurAAV-NC) group, and surgery + AAV9-CX3CR1-Ppargc1a (SurAAV-PGC1α) group. Following behavioral training, the SurAAV-PGC-1α group received bilateral hippocampal injections of AAV9-CX3CR1-Ppargc1a 21 days prior to surgery to ensure stable and cell-specific overexpression of PGC-1α in microglia. In contrast, the ConAAV-NC and SurAAV-NC groups were injected with empty AAV9 vectors as controls. Subsequently, the SurAAV-NC and SurAAV-PGC-1α groups underwent anesthesia and surgery. Behavioral assessments were conducted postoperatively. On postoperative day 3, hippocampal tissues were harvested for molecular and histological analyses. The experimental workflow is illustrated in Figure 8A. In parallel, *in vitro* experiments were conducted to further dissect the cell-autonomous effects of PGC-1α activation in microglia under surgery-related inflammatory conditions. BV-2 microglial cells were divided into three groups: vehicle control (NC), lipopolysaccharide plus isoflurane (LPS/ISO), and lipopolysaccharide plus isoflurane with ZLN005 pretreatment (LPS/ISO/ZLN). The LPS/ISO/ZLN group were pretreated with ZLN005 (15 μg/μL) for 24 h to pharmacologically activate PGC-1α, whereas the remaining groups received vehicle alone. Subsequently, the LPS/ISO and LPS/ISO/ZLN groups were exposed to 2.5% isoflurane and LPS (1 μg/μL) for an additional 24 h. To evaluate the impact of microglial PGC-1α activation on synaptic pruning, HT-22 neuronal cells were co-cultured with BV-2 cells at a 3:1 ratio to establish a neuron-microglia interaction model. Two co-culture conditions were generated: co-culture with LPS and isoflurane (Co-LPS/ISO) and co-culture with LPS, isoflurane, and ZLN005 pretreatment (Co-LPS/ISO/ZLN), following the same treatment paradigm applied to BV-2 cells. This co-culture system enabled direct assessment of how microglia-specific PGC-1α activation modulates mitochondrial metabolism and synaptic pruning under inflammatory stress. Cells were subsequently collected for downstream analyses.

### Behavior test

The Morris Water Maze (MWM) and Y-Maze tests are frequently used to evaluate cognitive function 37, 38. All behavioral tests were performed in a room with controlled temperature (20-23 °C) and humidity (30-50%) during the light phase (9:00 AM to 5:00 PM). The observers, unaware of the mice's group assignments, conducted the tests until completion. Before testing, each animal was allowed 15 minutes to acclimate the testing environment. The behavioral apparatuses were cleaned with 70% ethanol between sessions. Animal behaviors were recorded and analyzed using maze video tracking software (SMART V3.0.06, Panlab, Cornellà, Spain), and corresponding graphical responses were generated.

#### MWM test

The MWM test was performed to assess the cognitive abilities of mice, following protocols described in previous literature 39, with modifications to accommodate the conditions of mice after anesthesia and surgery. In the MWM test, the platform was positioned in the center of one quadrant, with a diameter of 10 cm and a height of 1 cm below the water surface. Briefly, all mice underwent a brief acclimation period to the water environment to ensure that they were able to swim. The mice received one swimming training session per day for four consecutive days. On the fifth day, anesthesia and surgery were performed on all mice, except those in the Con group. After surgery, mice continued swimming training for additional three days. The escape latency for each mouse was recorded, with a maximum duration of 60 seconds. A longer escape latency indicates deficiencies in learning and memory. The platform was removed on the seventh day to evaluate the number of crossings over the previous platform location and the time spent in the platform quadrant. Both a higher number of platform crossings and a longer time spent in the target quadrant indicate better memory capabilities that is, a higher cognitive level. The swimming speed of mice was recorded throughout to assess their motor capabilities.

#### Y-maze test

All groups of mice underwent a Y-maze test to assess working memory, as previously described 37, 40. The maze consists of three arms, each measuring 30×6×15 cm. During the trial, each mouse was placed in one arm (designated as arm A) and allowed to move freely between the arms for a total duration of 5 minutes. The software calculated the alternate transition rate as: actual alternate transition rate/maximum alternate transition rate×100%. The actual alternation value represented the number of times that the mouse consecutively entered three different arms. The maximum alternation value was determined by the total number of arm entries minus two. The Y-maze test is a widely used method to assess working memory in rodents and the SMART 3.0 software was employed to accurately quantify the spontaneous alternation behavior of mice. A lower alternate transition rate indicates impaired working memory.

### Single-nucleus RNA sequencing (snRNA-seq)

The SnRNA-seq was performed on the hippocampal tissues from the Con and Sur groups 3 days post-surgery to characterize the cell-type-specific transcriptional changes and microglial heterogeneity induced by anesthesia and surgery. Library preparation and sequencing were conducted on an Illumina NovaSeq X Plus platform by Gene Denovo Biotechnology Co., Ltd. (Guangzhou, China). Bioinformatic analyses were performed using the Omicsmart platform (https://www.omicsmart.com/). Briefly, the hippocampal tissues from three mice per group were dissociated using 1 mL Tryp-LE (Thermo Fisher Scientific, Waltham, Massachusetts, USA) at 37 °C for 35 min with gentle agitation every 5-8 min. The samples were washed three times with DPBS containing 2% FBS and gently triturated to obtain the single-nucleus suspensions. Approximately 13,000 nuclei per sample were processed using the 10× Genomics Chromium Single Cell 3′ system according to the manufacturer's protocol to generate cDNA libraries. The DoubletFinder was employed to calculate the probability of Gel Bead-in-Emulsion (GEM) containing multiple cells. The expected multiplet rate for each sample was then determined based on the relationship between the effective cell number (Cell Ranger filtered) and the multiplet rates provided by 10× Genomics. The sample-specific multiplet filtering thresholds were established, and multiple removal was performed sequentially. Data were aggregated and analyzed using the Seurat. The cells with mitochondrial gene contents ≥10% or gene counts < 300 or > 10,000 were excluded. Dimensionality reduction was performed using the PCA and visualized with the UMAP. the Findmarkers function with wilcox ranksum test were applied following the criteria: Min.pct = 0.25, *p* < 0.05 and |log₂ (fold change) | = 0.25 were used in finding DEGs. The canonical markers included *AQP4* (astrocytes), *P2RY12* (microglia), *Olig1* (OPCs), *GAD1/2* (inhibitory neurons), *SLC17A7* (excitatory neurons), and *MAG/MOBP* (oligodendrocytes). Clusters representing the same cell types were merged, and microglia were subjected to further sub-clustering. Differentially expressed genes (DEGs) were defined as those with an adjusted *p*-value < 0.05 and |log₂ (fold change) | > 0.25. Heatmaps, box plots, and dot plots were generated using Seurat and ggplot2. The pathway enrichment and Gene Ontology (GO) analyses were performed using the KEGG and annotations from the NCBI, the UniProt and the GO Consortium, with significance assessed via Fisher's exact test and FDR correction. The cell-cell communication networks were inferred using the CellChat (v1.6.1).

### Stereotaxic surgeries and injection of AAV

An AAV9 vector with a putatively microglia-specific CX3CR1 promoter was used to selectively drive overexpression of PGC-1α in the hippocampal microglia, following previously reported strategies for targeted microglial gene manipulation, including our previous work 41-43. Both the AAV-CX3CR1-Ppargc1a and AAV-CX3CR1-ZsGreen were constructed and produced by the HanBio Technology (Shanghai, China) to target and overexpress Ppargc1a in microglia, with the Gene ID 10891. The mice were anesthetized with 1.5% isoflurane, placed on a heating pad, and stabilized on a small-animal stereotactic apparatus (RWD Life Science Co., Ltd., Shenzhen, China). After sterilization, small holes were drilled bilaterally above the targeted injection site. AAV9-CX3CR1-Ppargc1α, AAV9-CX3CR1-Zsgreen, and empty AAV capsids (2 μL, 3.9×10¹² vg/mL) were bidirectionally injected into the hippocampus at a rate of 0.1µL/min using a syringe needle (33-gauge) and an automatic microinjection pump (KD Scientific, Holliston, MA, USA). The volume of the viral suspension injected into the unilateral hippocampus was 1 µL. Then the skull was fixed using dental cement. To ensure sufficient expression of the virus injected into hippocampus, PGC-1α overexpression or other virus were injected 3 weeks before anesthesia and surgery.

### Cell culture and co-culture system

BV-2 is a mouse brain-derived microglial cell line that retains key morphological and functional characteristics of primary microglia and that has been widely used as an effective *in vitro* substitute for primary microglial cultures 44. HT-22 is an immortalized mouse hippocampal neuronal cell line that has been widely used for the *in vitro* models to investigate neuronal function, synaptic plasticity, and mechanisms underlying learning and memory relevant to cognitive impairments 45. Both HT-22 cells (Procell, Wuhan, China) and BV2 cells (Jennio Biotech, Guangzhou, China) were cultured in 0.1 g/L poly-L-lysine coated culture flasks using Dulbecco's modified Eagle's medium (DMEM) (Procell) supplemented with 10% fetal bovine serum (Gibco, Thermo Fisher Scientific, Waltham, Massachusetts, USA) at 37 °C with 5% CO_2_. To establish an *in vitro* microglial model mimicking anesthesia- and inflammation-induced injury, BV-2 cells were treated with 1 μg/mL LPS (Escherichia coli, Sigma, St. Louis, MO, USA) for 24 h, as previously described 46, 47, followed by exposure to a gas mixture containing 2.5% isoflurane in air (2 L/min) for 4 h to induce cellular injury 48. Control cells received identical treatments but did not expose to LPS and isoflurane. Furthermore, based on previously established protocols 48, 49, a microglia-hippocampal neuron co-culture system was generated to assess the microglia-mediated synaptic pruning under anesthesia- and inflammation-related conditions. In this co-culture system, both HT-22 (1.5×10^4^/cm²) and BV-2 (0.5×10^4^/cm²) cells were seeded into the 12-well plates at a 3:1 ratio and cultured for 3 days in complete DMEM medium under optimal growth conditions. Isoflurane and LPS were subsequently applied to establish an *in vitro* model of anesthesia- and inflammation-induced injury. Notably, BV-2 and HT-22 cell lines are not listed as “commonly misidentified” in the International Cell Line Authentication Committee (ICLAC) database (https://iclac.org/databases/cross-contaminations/).

### Determination of mitochondrial membrane potential (MMP)

MMP was assessed using the JC-1 MMP Detection Kit (C2006, Beyotime, Shanghai, China). Briefly, 0.1 mL of purified mitochondria, containing 10-100 μg of total protein, was mixed with 0.9 mL of JC-1 staining solution. Fluorescent images were captured using an Olympus BX53 fluorescence microscope, with excitation at 485 nm and emission at 590 nm. The red/green fluorescence ratio was calculated, with a higher ratio indicating a higher MMP.

### Extracellular flux assays

Oxygen consumption rate (OCR) of BV-2 cells was measured using the Seahorse XF24 extracellular flux analyzer (Agilent, California, USA) with the Seahorse XFe Cell Mitochondrial Stress Test Kit (103010-100, Seahorse Bioscience, North Billerica, USA). BV-2 cells were plated in XF24 microplates at 2 × 10⁴ cells per well and prepared following the manufacturer's instructions. Prior to measurement, cells were washed three times with Seahorse assay medium (102353-100, Seahorse Bioscience) and incubated in a non-CO₂ incubator at 37 °C for 1 h. OCR was measured sequentially following the addition of three mitochondrial respiratory chain modulators: oligomycin (1.0 μM), carbonyl cyanide-4-(trifluoromethoxy) phenylhydrazone (FCCP, 1.0 μM), and a rotenone/antimycin A (Rot/AA) mixture (0.5 μM). This protocol allowed assessment of basal respiration, ATP-linked respiration, proton leak, maximal respiration, and spare respiratory capacity. Higher values of ATP-linked respiration, proton leak, maximal respiration, and spare respiratory capacity reflect greater mitochondrial energy metabolism potential.

### ATP level measurement

Hippocampal ATP level was measured using an ATP assay kit (HRK0910, Heruibio, Fuzhou, China). Briefly, hippocampal samples were lysed using ATP-specific lysis buffer, and the resulting extract was mixed with an ATP detection solution containing luciferase. Then, ATP levels were quantified using a microplate reader (SpectraMax iD3, Molecular Devices, California, USA).

### Immunofluorescence assay (IF)

Mice were deeply anesthetized and transcardially perfused with 0.9% saline followed by 4% paraformaldehyde. Brains were removed, sectioned (3 μm), permeabilized with 4% paraformaldehyde, and blocked with 3% BSA for 1 h. Sections were then incubated overnight at 4 °C with primary antibodies. CD68 (28058-1-AP, Proteintech, Wuhan, China), CX3CR1 (13885-1-AP, Proteintech), PSD-95 (20665-1-AP, Proteintech), PSD-95 (M1511-4, HuaBio, Huangzhou, China), Synaptophysin (A19122, ABclonal, Wuhan, China) and IBA-1 81728-1-RR, Proteintech). After washing with PBS, sections were incubated for 1 h at room temperature with the appropriate secondary antibodies. The secondary antibodies used were iFluor™ 488 Conjugated Goat anti-rabbit IgG polyclonal antibody (HA1121, HuaBio), iFluor™ 594 Conjugated Rabbit anti-Goat IgG polyclonal antibody (HA1132, HuaBio), iFluor™ 594 Conjugated Goat anti-mouse IgG polyclonal antibody (HA1126, HuaBio), and iFluor™ 488 Conjugated Goat anti-mouse IgG polyclonal antibody (HA1121, HuaBio). The images were observed and acquired using an Olympus BX53 microscope (Olympus Corporation, Japan) with the standardized acquisition settings (laser power, detector gain, exposure, pinhole) calibrated to prevent saturation. For each region of interest area (ROI), 10 coronal sections with a 30-μm thickness per animal were analyzed at the fixed intervals. Fields of view in the hippocampal CA1 region were captured using a grid-based scheme. A standard slide was used for daily calibration, and sample imaging order was randomized across groups to minimize the potential drift. The expression level of interested markers including CD68 and CX3CR1 were assessed using the predefined thresholds by negative controls, with consistent intensity and size criteria across images. Fluorescence thresholds were determined by [mean±SD] and maintained within batches. The Fiji's 'Colocalization' tool was used for identifying and counting fluorescent overlaps as positive cells. Image acquisition, ROI selection, and quantification were conducted by the investigators who were blinded to experiment conditions. Microglial phagocytosis of synaptic PSD-95 was analyzed with the IMARIS software (Bitplane, Zürich, Switzerland). The "Surface" function was used to reconstruct a three-dimensional surface of microglia, while the "Spots" function was applied to identify PSD-95 spots. The co-localization analysis was performed to quantify the number of PSD-95-positive spots fully contained within the microglial surface.

### Golgi-Cox staining and Sholl analysis

The Golgi-Cox staining is a classical technique for visualizing complete neuronal morphology, including soma, axons, dendrites, and dendritic spines 50. The Golgi-Cox staining kit (GP1152, Servicebio Technology, Wuhan, China) was used to stain brain tissues three days post-surgery. After deep anesthesia with 2% sodium pentobarbital, mice were sacrificed, and brains were rinsed with double-distilled water. Brains were immersed in Solutions A and B (Golgi-Cox kit, GP1152, Servicebio, Wuhan, China) for 14 days, followed by Solution C for 7 days. Samples were sectioned at 100 µm using a vibrating microtome, mounted on gelatin-coated slides, dehydrated with alcohol, cleared with xylene, and cover-slipped. Neuronal structures were imaged using an Olympus BX51 microscope. Neuronal morphology and dendritic complexity were analyzed using the Sholl plugin in ImageJ (NIH, Bethesda, Maryland, USA). Grayscale images of isolated neurons were skeletonized, and dendrites and pseudopodia were quantified. The maximum number of intersections was used as an indicator of neuronal complexity.

### Transmission electron microscopy (TEM)

TEM was used to assess synaptic structures, including synaptic vesicles, PSD and synaptic counts. Mice were deeply anesthetized and transcardially perfused with PBS. The hippocampus was dissected and immediately immersed in TEM fixative. Tissue samples were cut into ~1 mm³ blocks, post-fixed in 1% osmium tetroxide for 2 h at room temperature, and rinsed three times for 15 min each in 0.1 M PBS. Samples were dehydrated through a graded ethanol series, embedded in resin, and sectioned at 60-80 nm using an ultramicrotome. Sections were mounted on 150-mesh copper grids coated with formvar film, stained with 2% uranyl acetate for 8 min, rinsed with 70% ethanol and ultrapure water, and counterstained with 2.6% lead citrate for 8 min. Images were acquired by TEM and analyzed with ImageJ. Higher synaptic vesicle numbers, synaptic counts, and thicker PSDs indicate better synaptic integrity.

### Western blot (WB) analysis

WB analysis was conducted to examine relative protein expression in the hippocampus. Hippocampal tissues were lysed in RIPA buffer on ice for 30 min and centrifuged at 12,000 g for 10 min at 4 °C. Protein concentrations were determined using a bicinchoninic acid (BCA) assay kit (EpiZyme, Shanghai, China). Equal amounts of protein (30 µg) were separated by 10% or 12.5% SDS-PAGE and transferred onto PVDF membranes (Millipore, Billerica, MA, USA). Membranes were washed three times with TBST and blocked with 2% BSA for 2 h at room temperature, followed by overnight incubation at 4 °C with primary antibodies against PGC1-α (A12348, ABclonal), NRF-1 (A3251, ABclonal), TFAM (A3173, ABclonal), COX IV (11242-1-AP, Proteintech), ATP5A1 (14676-1-AP, Proteintech), PSD-95 (20665-1-AP, Proteintech), Synaptophysin (17785-1-AP, Proteintech), NMDAR1 (ET1703-75, HuaBio), NMDAR2A (ET1704-80, HuaBio), NMDAR2B (HA722284, HuaBio), and β-actin (R1207-1, HuaBio). After washing, membranes were incubated with the appropriate secondary antibodies. Protein bands were visualized using enhanced chemiluminescence (ECL; MeilunBio, Dalian, China), imaged with a ChemiDoc system (Bio-Rad Laboratories), and quantified using ImageLab software (Bio-Rad Laboratories). Results are presented as relative band density values.

### Statistical analysis

All data were statistically analyzed using GraphPad Prism 9.5 (GraphPad Software, San Diego, CA, USA). Statistical methods were selected based on data characteristics, including normality tests, Student's t-test, and one-way ANOVA. Normality tests were applied to determine data distribution. Normally distributed data were presented as mean ± standard deviation (SD). Student's t-tests compared two groups, while one-way ANOVA followed by Bonferroni post hoc comparisons was used for multiple group comparisons. Repeated measures two-way ANOVA, followed by Bonferroni post hoc comparisons, was employed for multiple groups at different time points. Non-normally distributed data were presented as median (interquartile range) and analyzed using the Kruskal-Wallis test for multiple groups. These methods ensured robust and appropriate data analysis. A *p*-value of less than 0.05 (two-tailed) was considered statistically significant.

## Results

### Anesthesia and surgery induced cognitive dysfunction and impaired synaptic plasticity in aged mice

Figure 1A illustrated the experimental timeline. Cognitive performance was evaluated using the MWM and Y-maze tests. During the pre-surgery training phase of the MWM (days 1-4), the Con and Sur groups displayed similar performance (Figure 1B, C). However, in the post-surgery testing phase (days 7 and 8), the Sur group required a significantly prolonged time to reach the hidden platform compared to the Con group (Figure 1C, D). On day 8, the Sur group also showed fewer platform crossings and spent less time in the target quadrant than the Con group (Figure 1E). No significant difference in swimming speed was observed between Con and Sur groups (Figure 1F). In the subsequent Y-maze spontaneous alternation test to assess spatial and working memory after the MWM, behavioral trajectories were recorded and alternation rates were quantified (Figure 1G). The Sur group exhibited a significantly lower spontaneous alternation rate than the Con group (Figure 1H).

To investigate the effects of anesthesia and surgery on the hippocampal synaptic plasticity, Western blot was firstly performed to examined the expression levels of hippocampal synaptic proteins, including postsynaptic density protein 95 (PSD-95), synaptophysin (SYN), and N-methyl-D-aspartate receptor (NMDAR) subunits (NMDAR1, NMDAR2A and NMDAR2B). As shown in Figure 1I and Figure S2, the expression levels of hippocampal PSD-95, SYN, NMDAR1, NMDAR2A and NMDAR2B were significantly decreased in the Sur group compared with the Con group, indicating a marked synaptic loss following anesthesia and surgery. To further evaluate synaptic plasticity, we performed the immunofluorescence staining for PSD-95 and SYN, TEM and Golgi-Cox staining combined with Sholl analysis. The Immunofluorescence results showed significantly reduced PSD-95 and SYN signal intensities in the Sur group compared to the Con group (Figure 1L-N), with decreased co-localization of PSD-95 with SYN in the hippocampal CA1 region (Figure 1O). TEM revealed notable synaptic ultrastructural changes in the Sur group, including decreased postsynaptic density thickness, synaptic vesicles and synapses, and disrupted postsynaptic membrane continuity (Figure 1P-S). In addition, Sholl analysis demonstrated significant reductions in dendritic spine density and in the number of maximum intersection points in the Sur group compared with the Con group (Figure 1T-W). Collectively, these findings indicate that anesthesia and surgery induce substantial hippocampal synaptic damage and cognitive dysfunction in aged mice.

### The snRNA-seq revealed alterations in microglial phagocytosis subtype following anesthesia and surgery

**Figure 2:**
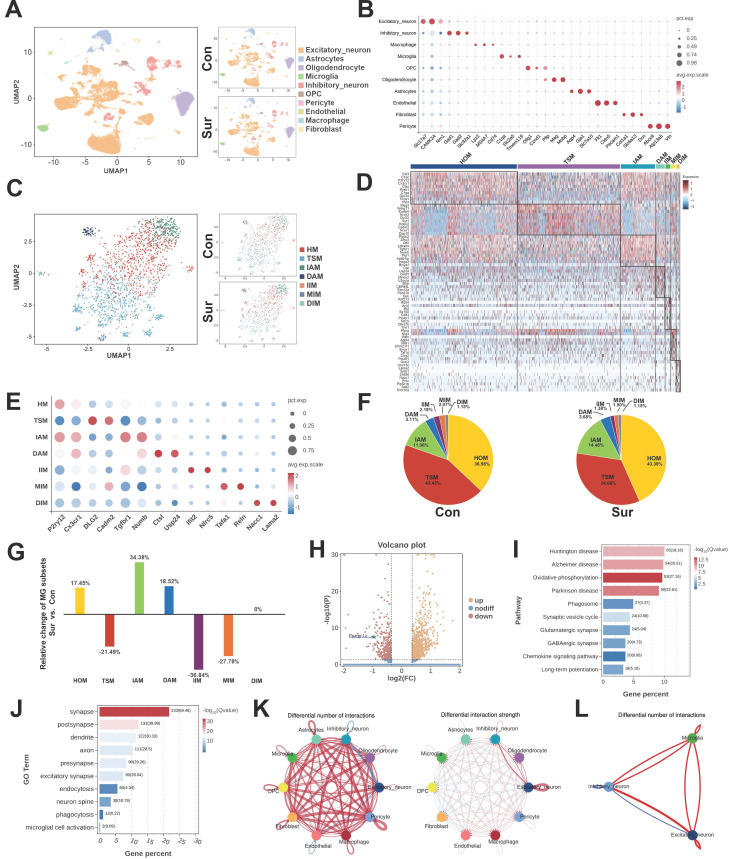
** The SnRNA-seq analysis of the hippocampus in aged mice after anesthesia and surgery.** (A) UMAP dimensionality reduction maps of ten cell types in hippocampus; (B) Dot plot showed specific highly expressed genes in each cell type of hippocampus; (C) Secondary clustering identified four microglial subpopulations with distinct transcriptomic profiles; (D) Heatmap showed the top ten DEGs of each subpopulation; (E) Dot plot showed the expression of the marker genes in different subpopulations; (F) Pie charts showed the proportion of microglial subpopulations relative to the total microglia; (G) Bar graphs showed the relative change in the Sur group compared with the Con group, calculated as (proportion in the Sur group- proportion in the Con group)/proportion in the Con group; (H) The volcanic plot illustrated the expression of microglial Ppargc1a in the Sur group compare to the Con group; (I) KEGG enrichment analysis of hippocampal microglia; (J) GO enrichment analysis of hippocampal microglia; (K) Comparisons for the changes of cell-cell interactions in all pairs of cells between the Sur and Con groups. Red edges represented increased signalings in the Sur group, blue edges represented increased signalings in the Con group; (L) Comparisons for the changes of cell-cell interactions in all pairs of microglia, inhibitory neuron and excitatory neuron between the Sur and Con groups.

Hippocampal tissues from the Con and Sur mice were collected three days postoperatively for snRNA-seq, yielding 48,814 cells. Unsupervised clustering identified ten major cell populations, including excitatory and inhibitory neurons, microglia, astrocytes, oligodendrocytes, OPCs, endothelial cells, pericytes, macrophages, and fibroblasts (Figure 2A). A heatmap showing the top DEGs for each cell type (Figure 2B). In the microglia, representative DEGs included *Tmem119*, *Slc2a5*, and *C1qa*, which are associated with microglial activation, complement signaling, and neuron-microglia interactions. Given the close association between microglial phagocytic activity and synaptic pruning, we performed sub-clustering of 1,737 hippocampal microglia, identified seven transcriptionally distinct subpopulations (Figure 2C). These included homeostatic microglia (HOM, 40.18%), transition-state microglia (TSM, 38.74%), inflammation-associated microglia (IAM, 12.95%), disease-associated microglia (DAM, 3.40%), and three inhibitory subtypes, such as inflammation inhibitory microglia (IIM, 1.78%), metabolism-associated inhibitory microglia (MIM, 1.78%), and disease-associated inhibitory microglia (DIM, 1.15%). A heatmap of the top four DEGs in each subpopulation highlights their distinct transcriptional signatures (Figure 2D, E).

The comparative analysis revealed significant shifts in the microglial subpopulation compositions following anesthesia and surgery (Figure 2F, G). Compared with the Con group, the Sur group showed the increased proportions of HOM (36.98% to 43.38%), IAM (11.06% to 14.48%) and DAM (3.11% to 3.68%), with decreased proportions of TSM (43.43% to 34.06%), IIM (2.19% to 1.38%) and MIM (2.07% to 1.50%). The proportion of DIM remained largely unchanged. Notably, the expression of *Ppargc1a*, encoding the mitochondrial regulator PGC-1α, was significantly downregulated in the microglia from the Sur group (Figure 2H), suggesting an impaired mitochondrial metabolic capacity. Consistently, the KEGG pathway analysis revealed enrichment in the pathways related to oxidative phosphorylation, phagosome formation, synapse regulation, chemotaxis, and neurodegenerative diseases (Figure 2I). The GO analysis further demonstrated the upregulations of biological processes associated with synapses, dendritic spines, endocytosis, and phagocytosis in the microglia from the Sur group (Figure 2J). Finally, the CellChat analysis demonstrated an enhanced intercellular communication following surgery, with increased signaling from the microglia to excitatory and inhibitory neurons (Figure 2K, L). Collectively, these findings suggest that in aged mice, anesthesia and surgery promote a shift of hippocampal microglia toward a phagocytic and metabolically reprogrammed phenotype potentially driven by PGC-1α downregulation, contributing to excessive synaptic pruning and POCD.

### Anesthesia and surgery promoted microglial phagocytosis and synaptic pruning in the hippocampus of aged mice

**Figure 3:**
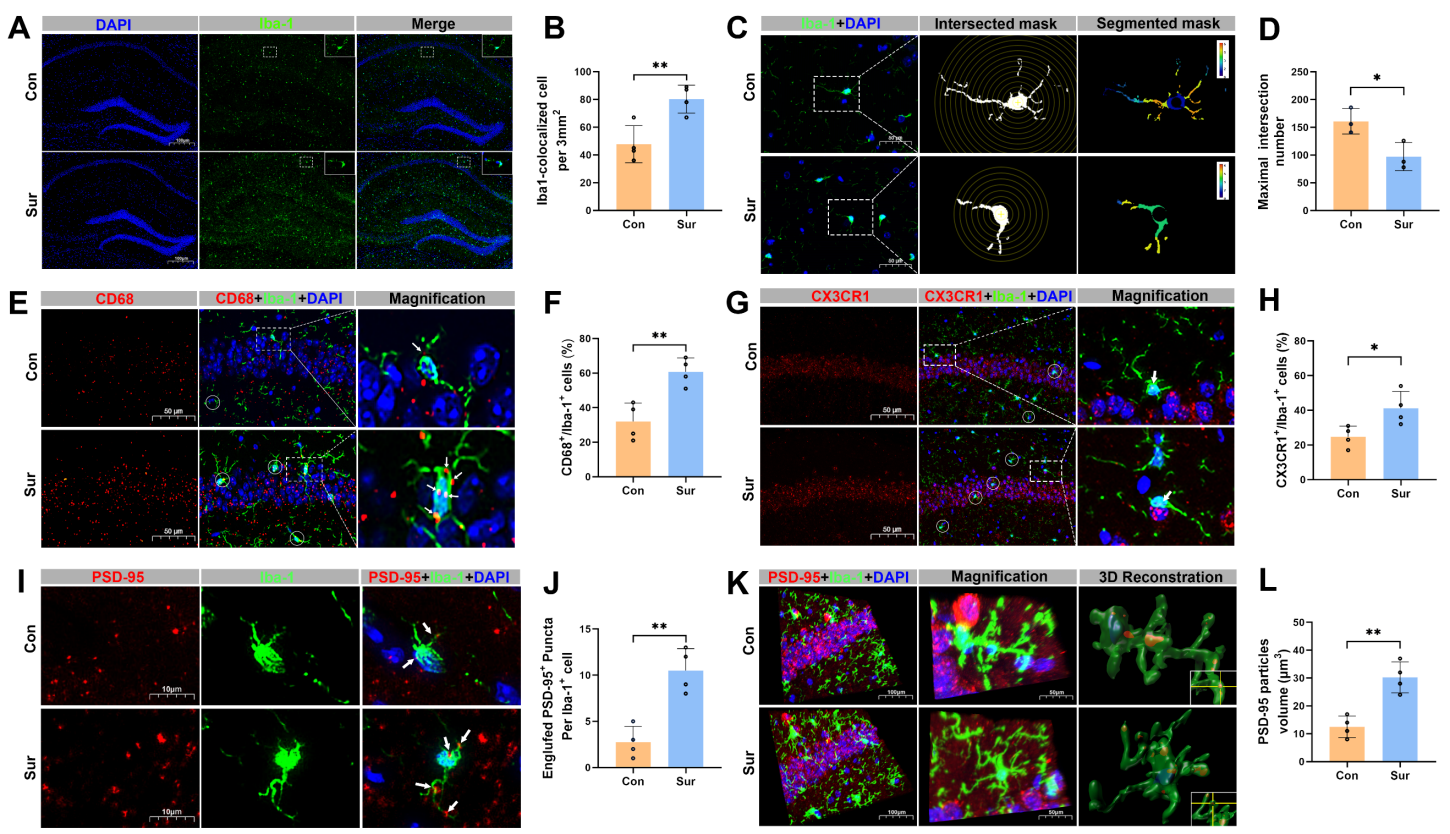
**Anesthesia and surgery promoted microglial phagocytosis and synaptic pruning in the hippocampus of aged mice.** (A) Representative immunofluorescence images of microglial marker Iba-1 in the hippocampus; n=5; (B) Quantificative analysis for the number of Iba-1^+^ cells per 5000 μm^2^ in the hippocampus, n=4; (C) Representative images for Golgi-cox staining with Sholl analysis of the iba-1^+^ cells in the hippocampal CA1 region; (D) Quantificative analysis for the morphological changes of microglia through maximum number of intersection points in the hippocampal CA1 region, n=5; (E) Representative double-immunofluorescence images of CD68 (red) and Iba-1 (green) in the hippocampal CA1 region; (F) Quantificative analysis for CD68^+^/Iba-1^+^ cells in the hippocampal CA1 region, n=4; (G) Representative double-immunofluorescence images of CX3CR1 (red) and Iba-1 (green) in the hippocampal CA1 region; (H) Quantificative analysis for CX3CR1^+^/Iba-1^+^ cells in the hippocampal CA1 region, n=4; (I) Representative double-immunofluorescence images of PSD-95 (red) and Iba-1 (green) in the hippocampal CA1 region; (J) Quantificative analysis for engulfed PSD-95^+^ puncta co-located with Iba-1 per cells in the hippocampal CA1 region, n=4; (K) Representative double-immunofluorescence with 3D surface reconstruction images of PSD-95 (red) and Iba-1 (green) in the hippocampal CA1 region; (L) Quantificative analysis for PSD-95 particles volume in Iba-1^+^ cells in the hippocampal CA1 region, n=4; Data are presented as the mean ± SD. Student's t-test was used to obtain *p*-value. *p <0.05, **p < 0.01.

Immunostaining of the hippocampal CA1 region revealed that Iba-1 expression was significantly increased in the Sur group compared with the Con group (Figure 3A, B). Sholl analysis of microglia morphology demonstrated a reduction in branching complexity in Sur mice, as indicated by a decreased number of microglial intersections, which reflects a shift toward an activated, phagocytic state and suggests associated synaptic retraction (Figure 3C, D). Co-staining for Iba-1 and the phagocytic marker CD68 showed elevated co-localization in the Sur mice, further supporting increased microglial phagocytosis after anesthesia and surgery (Figure 3E, F). To explore the potential microglia-neuron interactions, the expression of CX3CR1 and its co-localization with Iba-1 in the CA1 region were examined. A significant increase in CX3CR1/Iba-1 co-localization was observed in the Sur group compared to the Con group, suggesting an enhanced microglial recruitment or adhesion to neuronal elements (Figure 3G, H). Next, the microglia-mediated synaptic engulfment was further evaluated by assessing co-localization of Iba-1 with the postsynaptic marker PSD-95. The 3D reconstruction showed more PSD-95 signal within Iba-1-positive microglia in the Sur group than in the Con group (Figure 3I-L), indicating an increased microglial engulfment of synaptic components. All of these support that anesthesia and surgery activate microglia in the hippocampus, enhance their phagocytic activity, and promote direct microglia-synapse interactions, leading to an increased synaptic pruning and an aggravated synaptic damage.

### Anesthesia and surgery reduced PGC-1α expression and caused mitochondrial dysfunction *in vivo* and *in vitro*

**Figure 4:**
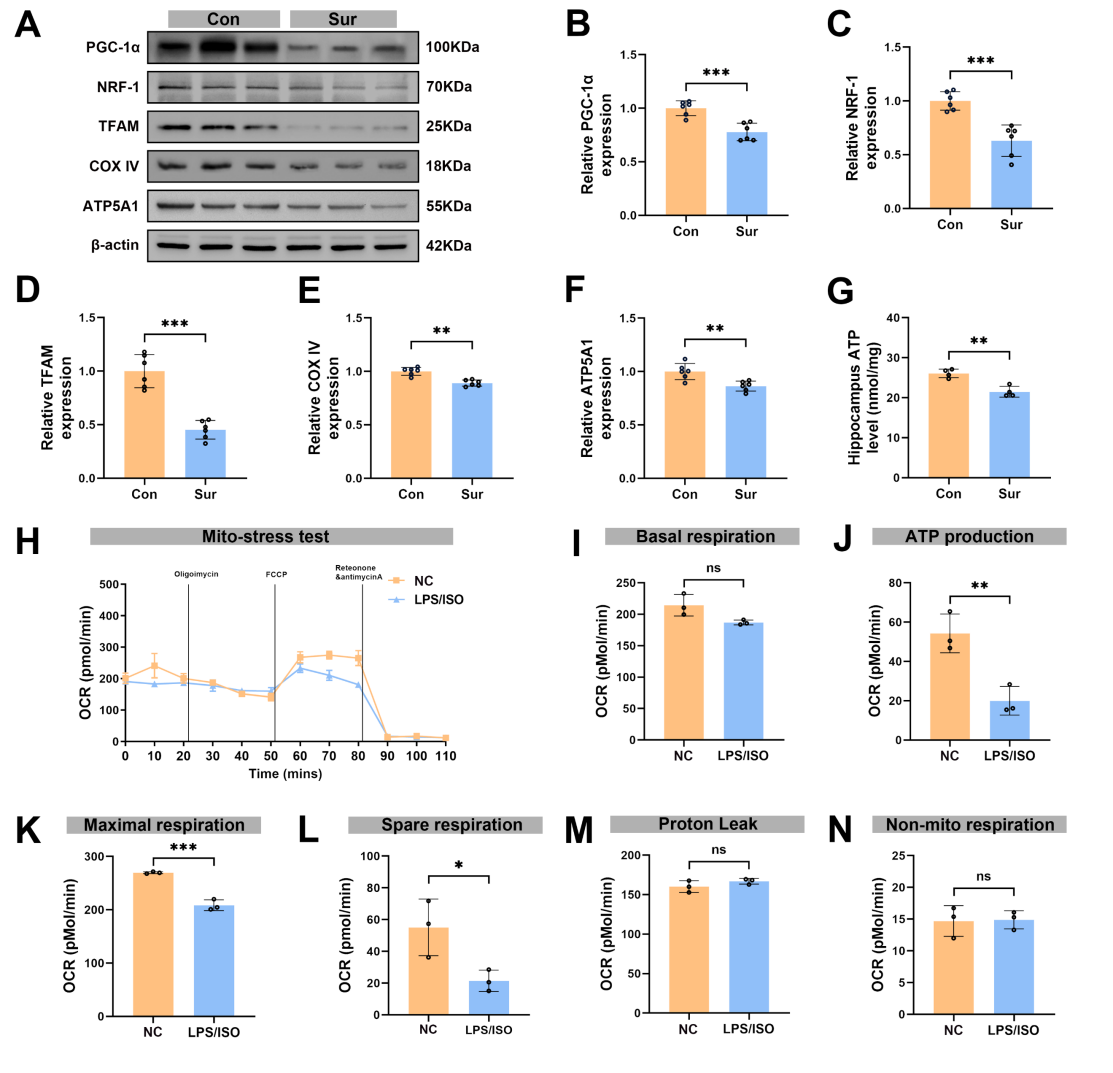
** Anesthesia and surgery reduced PGC-1α expression and caused mitochondrial dysfunction *in vivo* and *in vitro*.** (A) Representative Western blots of PGC-1α, NRF-1, TFAM, COX IV and ATP5A1 in the hippocampaus of aged mice; (B-F) The relative expression levels of PGC-1α, NRF-1, TFAM, COX IV and ATP5A1 in the hippocampus, n=6; (G) Quantificative analysis for hippocampal ATP levels, n=4; (H) Diagram of OCR in the Seahorse XF mitochondrial stress test of BV-2 *in vitro* modeling, n=3; (I) Quantificative analysis for basal respiration, n=3; (J) Quantificative analysis for ATP production in NC group, n=3; (K) Quantificative analysis for maximal respiration, n=3; (L) Quantificative analysis for spare respiration capacity, n=3; (M) Quantificative analysis for proton leak capacity, n=3; (N) Quantificative analysis for non-mitochondrial respiration, n=3; Data are presented as the mean ± SD. Student's t-test was used to obtain *p*-value. *p <0.05, **p < 0.01, ***p < 0.001, ns, not significant.

We next assessed the impacts of anesthesia and surgery on mitochondrial regulators and function. The Western blot analysis showed a significant decrease in hippocampal PGC-1α expression on post-surgery day 3 (Figure 4A, B), with marked reductions in expression levels of NRF-1 and TFAM (Figure 4A, C, D). In addition, the abundance of mitochondrial markers COX IV and ATP synthase F1 subunit α (ATP5A1) in the hippocampus was lower in the Sur group than in the Con group, and total ATP content in the hippocampus was also significantly reduced in the Sur group (Figures 4A, E-G). These changes point to a breakdown in mitochondrial energy metabolism after anesthesia and surgery. To further assess mitochondrial function of microglia, BV-2 microglial cells were exposed to inflammatory conditions and isoflurane (LPS + isoflurane) to mimic the *in vivo* surgical and anesthesia insults, and then the OCR was measured (Figure 4H). Mitochondrial stress tests showed that ATP production (Figure 4J), maximal respiration (Figure 4K) and spare respiratory capacity (Figure 4L) were significantly reduced after treatments, whereas basal respiration (Figure 4I), proton leak (Figure 4M), and non-mitochondrial respiration (Figure 4N) were not changed, demonstrating a pronounced impairment of mitochondrial respiration in microglia. Together these data indicate that anesthesia and surgery disrupt mitochondrial homeostasis in the hippocampus, reducing PGC-1α and key mitochondrial biogenesis factors. These events likely contribute to the metabolic dysfunction observed after surgery.

### Enhanced PGC-1α expression improved mitochondrial function and alleviated microglial synaptic pruning *in vivo* and *in vitro*

**Figure 5:**
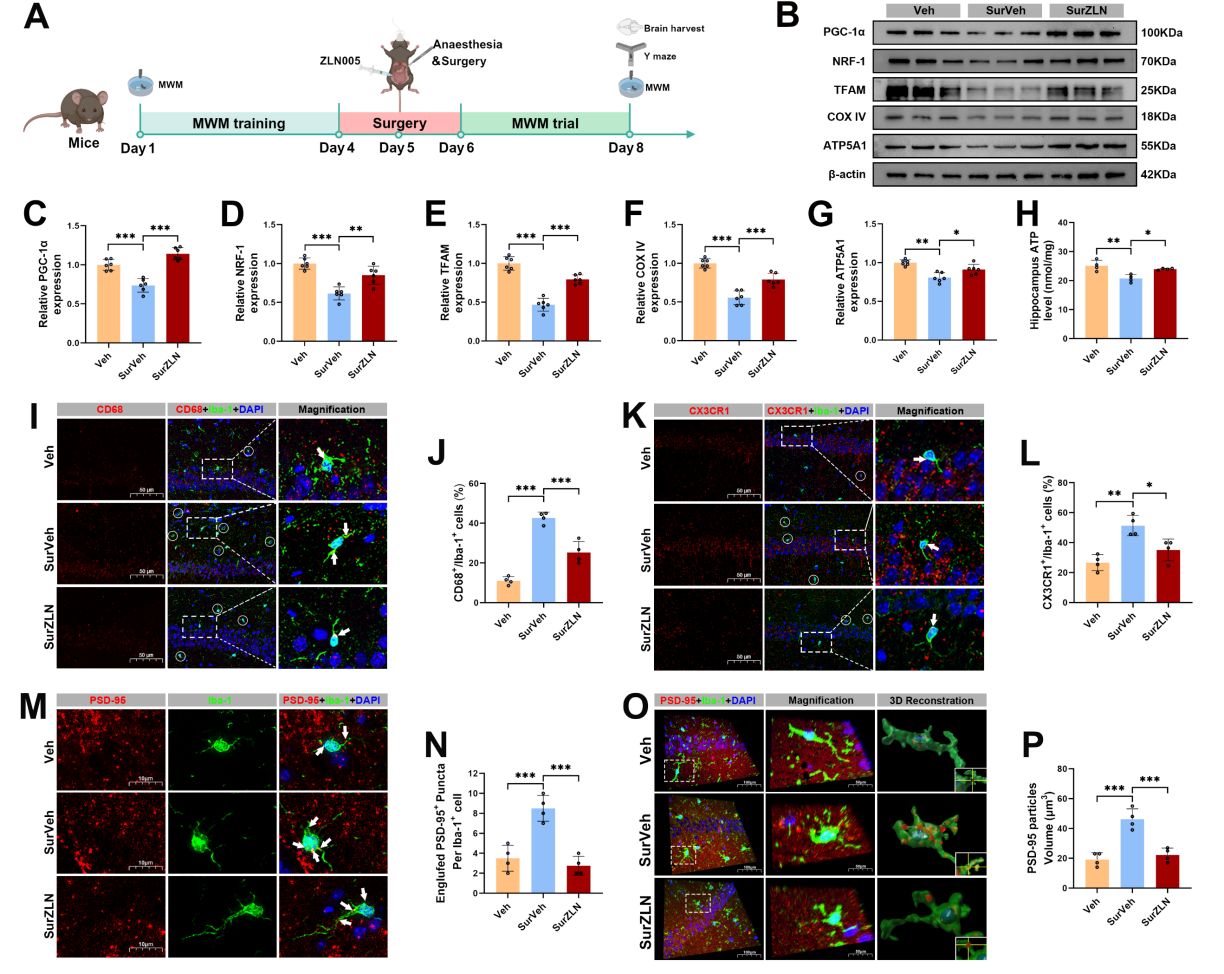
** Effects of ZLN005 treatment on mitochondrial function and hippocampal synaptic pruning following anesthesia and surgery in aged mice.** (A) Experimental flowchart with applying ZLN005; (B) Representative Western blots of PGC-1α, NRF-1, TFAM, COX IV and ATP5A1 in the hippocampus of aged mice; (C-G) The relative expression levels of PGC-1α, NRF-1, TFAM, COX IV and ATP5A1 in the hippocampus, n=6; (H) Quantificative analysis for hippocampal ATP levels, n=4; (I) Representative double-immunofluorescence images of CD68 (red) and Iba-1 (green) in the hippocampal CA1 region; (J) Quantificative analysis for CD68^+^/Iba-1^+^ cells in the hippocampal CA1 region, n=4; (K) Representative double-immunofluorescence images of CX3CR1 (red) and Iba-1 (green) in the hippocampal CA1 region; (L) Quantificative analysis for CX3CR1^+^/Iba-1^+^ cells in the hippocampal CA1 region, n=4; (M) Representative double-immunofluorescence images of PSD-95 (red) and Iba-1 (green) in the hippocampal CA1 region; (N) Quantificative analysis for engulfed PSD-95^+^ puncta co-located with Iba-1 per cells in the hippocampal CA1 region, n=4; (O) Representative double-immunofluorescence with 3D surface reconstruction images of PSD-95 (red) and Iba-1 (green) in the hippocampal CA1 region; (P) Quantificative analysis for PSD-95 particles volume in Iba-1^+^ cells in the hippocampal CA1 region, n=4; Data are presented as the mean ± SD. One-way ANOVA followed by Bonferroni's multiple comparisons test was used to obtain *p*-value. *p <0.05, **p < 0.01, ***p < 0.001.

We administered the PGC-1α activator ZLN005 one hour before anesthesia and surgery (Figure 5A). In the SurZLN group, hippocampal PGC-1α expression was significantly increased compared with the SurVeh group (Figure 5B, C). Correspondingly, the expression levels of mitochondrial biogenesis regulators NRF-1 and TFAM were restored (Figure 5B, D, E). The expression levels of mitochondrial markers COX IV and ATP5A1 were upregulated, and hippocampal ATP content was significantly higher in the SurZLN mice than in SurVeh mice (Figure 5B, F-H), indicating a rescued mitochondrial energy metabolism Next, immunostaining revealed that ZLN005 treatment markedly reduced the co-localization of CD68 and CX3CR1 with Iba-1 in the hippocampal microglia of SurVeh mice (Figure 5I-L), suggesting attenuated microglial phagocytosis. In addition, 3D reconstruction of immunofluorescence staining for Iba-1 and PSD-95 showed significantly less PSD-95 engulfment by Iba-1-positive microglia in the SurZLN group than in the SurVeh group (Figure 5M-P), indicating a reduced synaptic pruning.

**Figure 6:**
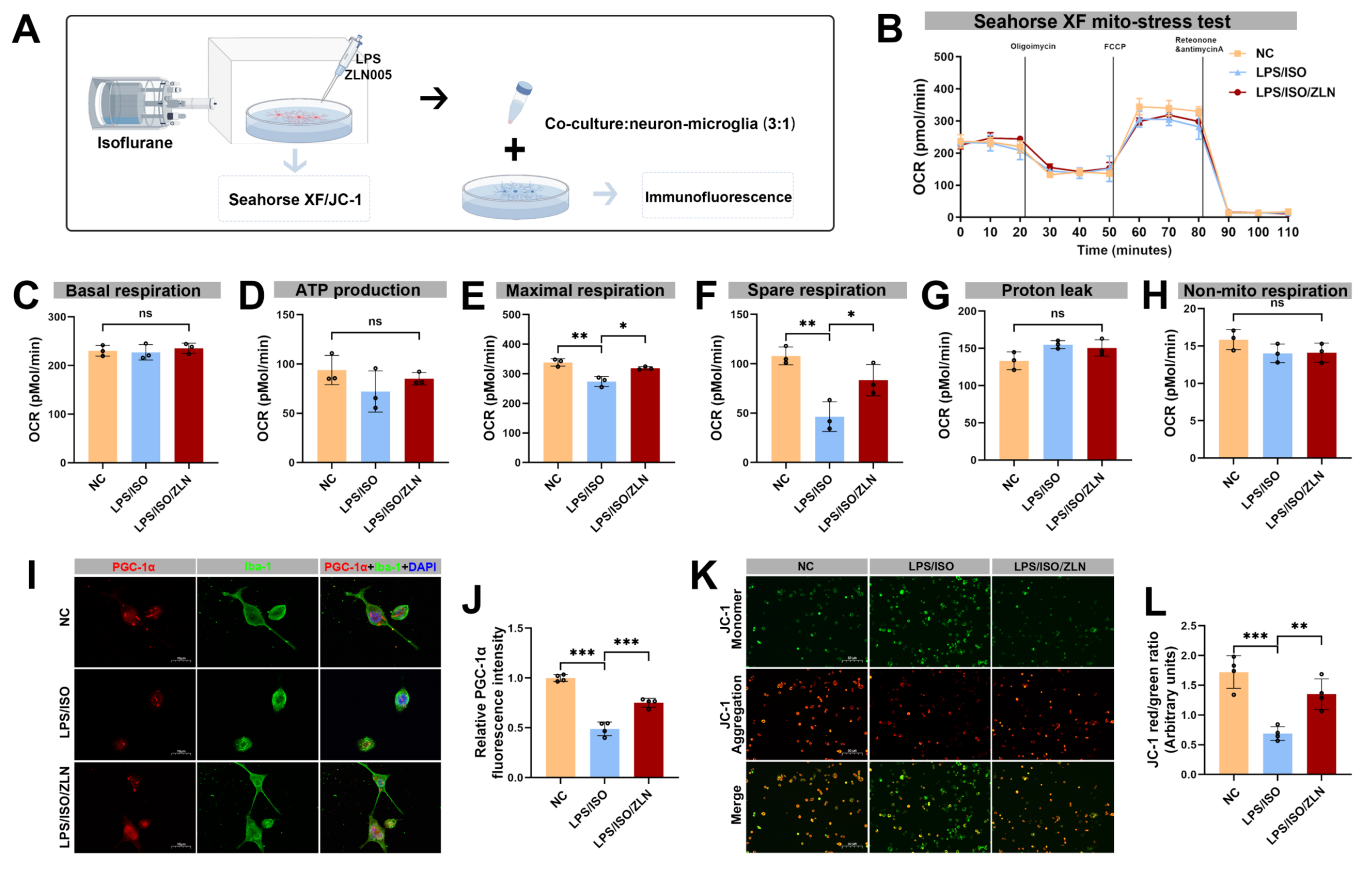
** Effects of ZLN005 treatment on mitochondrial function in BV-2 cells following isoflurane and LPS exposure.** (A) Schematic diagram of the co-culture of BV-2 and HT-22 cells *in vitro*; (B) Diagram of OCR in BV-2 cells from Seahorse XF mitochondrial stress test after ZLN005 treatment, n=3; (C) Quantificative analysis for basal respiration, n=3; (D) Quantificative analysis for ATP production, n=3; (E) Quantificative analysis for maximal respiration, n=3; (F) Quantificative analysis for spare respiration capacity, n=3; (G) Quantificative analysis for proton leak capacity, n=3; (H) Quantificative analysis for non-mitochondrial respiration, n=3; (I) Representative double-immunofluorescence images of PGC-1α (red) and Iba-1 (green) of BV-2 cells line, n=4; (J) Fluorescence intensity of PGC-1α in Iba-1^+^ cells, n=4; (K) Representative JC-1 staining image of BV-2 cells line, n=4; (L) MMP measured as the ratio of red to green fluorescence intensity from, n=4; Data are presented as the mean ± SD. One-way ANOVA followed by Bonferroni's multiple comparisons test was used to obtain *p*-value. *p <0.05, **p < 0.01, ***p < 0.001, ns, not significant.

In the microglial BV-2 cells exposed to isoflurane and LPS to mimic surgery-related insults (Figure 6A), ZLN005 treatment compared with the LPS/ISO group effectively restored mitochondrial respiration (Figure 6B), namely, ATP production, maximal respiration, and spare respiratory capacity were significantly increased (Figure 6D-F), whereas there were no significant differences among groups in basal respiration (Figure 6C), proton leak (Figure 4G), and non-mitochondrial respiration (Figure 6H). ZLN005 treatment not only enhanced microglial PGC-1α expression but also suppressed microglial activation, as demonstrated by increased PGC-1α immunoreactivity within Iba-1⁺ cells, indicating a shift toward a less activated microglial phenotype following ISO/LPS exposure (Figure 6I, J). Furthermore, ZLN005 treatment preserved mitochondrial membrane potential, as shown by JC-1 staining (Figure 6K, L). In a co-culture system of BV-2 and neuronal HT-22 cells, LPS/ISO treatment enhanced microglial phagocytosis of PSD-95, which was significantly reduced by ZLN005 treatment, further supporting the role of PGC-1α in inhibiting microglia-mediated synaptic engulfment (Figure S1A, B).

### Enhanced microglial PGC-1α expression reversed hippocampal synaptic damage and cognitive dysfunction after anesthesia and surgery in aged mice

**Figure 7:**
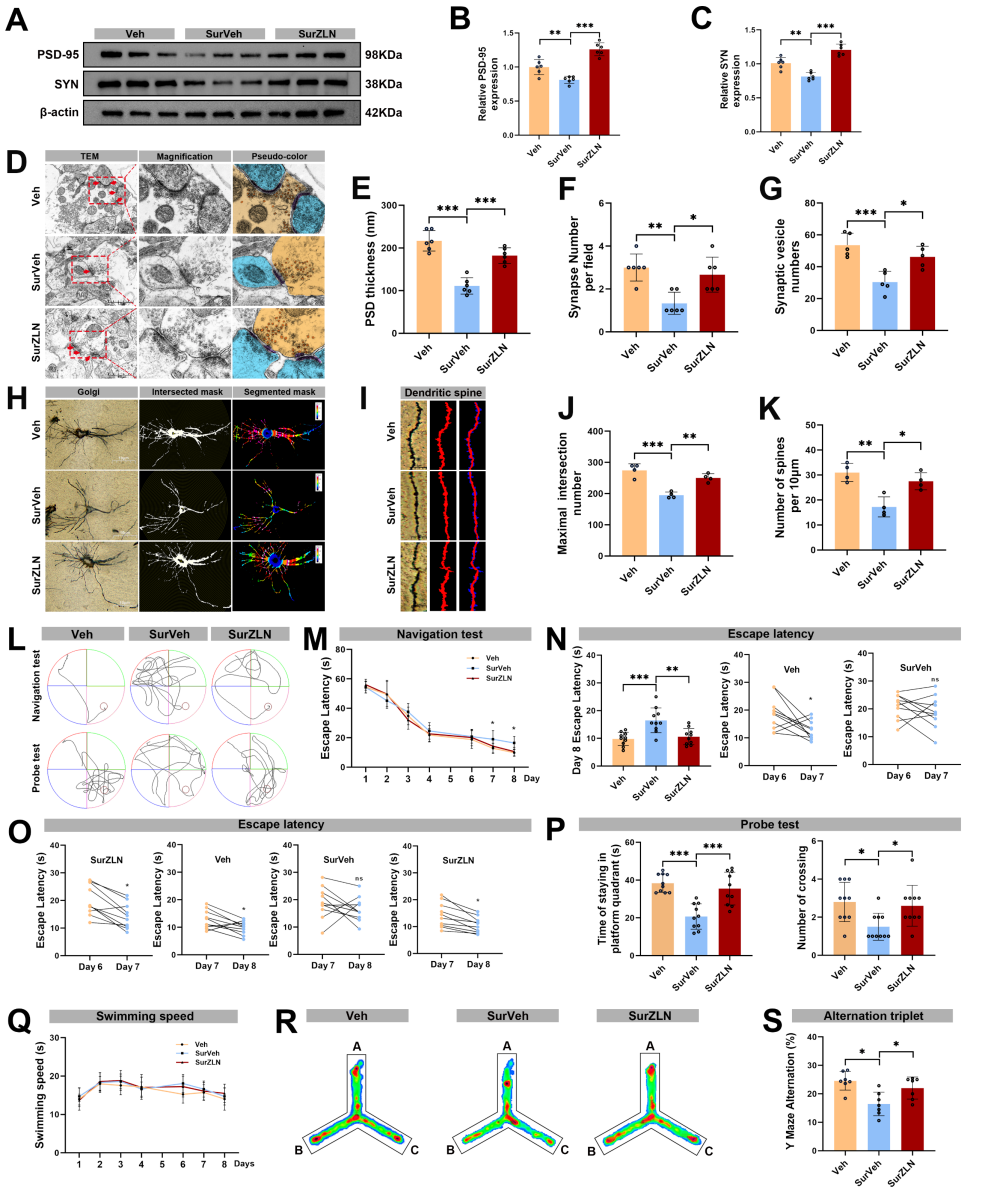
** Effects of ZLN005 treatment on synaptic plasticity and cognitive function following anesthesia and surgery in aged mice.** (A) Representative Western blots of PSD-95 and SYN in the hippocampus of aged mice; (B) The relative expression levels of PSD-95 and SYN in the hippocampus, n=6; (D) Representative TEM images of synapses in the hippocampal CA1 region; (E-G) Quantificative analysis for the thickness of postsynaptic density, synaptic vesicles, and synapse number in the hippocampal CA1 region by TEM, n=6; (H) Representative Golgi-cox staining images of neurons in the hippocampal CA1 region; (I) Representative Golgi-cox staining with Sholl analysis images of neurons in the hippocampal CA1 region; (J) Quantificative analysis for maximum number of intersection points in the hippocampal CA1 region, n=4; (K) Quantificative analysis for the number of dendritic spines, n=4; (L) Trajectories of swimming in the MWM test; (M-O) Escape latency in the MWM test, n=10; (P) Time of staying on the platform and number of crossing the platform in the MWM test, n=10; (Q) Swimming speed in the MWM test, n=10; (R) Heatmap of paths in the Y-maze test; (S) Y-maze spontaneous alternation rate, n=7. Data are presented as the mean ± SD. One-way ANOVA followed by Bonferroni's multiple comparisons test was used to obtain *p*-value. *p <0.05, **p < 0.01, ***p < 0.001, ns, not significant.

To test whether restoring PGC-1α expression could reverse synaptic damage, we measured expression levels of hippocampal synaptic proteins PSD-95 and SYN by Western blot. The expression levels of PSD-95 and SYN were significantly higher in the SurZLN group than in the SurVeh group (Figure 7A-C). The TEM revealed that the SurZLN mice had increased PSD thickness, synaptic vesicles and number of synapses compared with the SurVeh mice (Figure 7D-G). The Golgi-Cox staining with Sholl analysis further showed that the SurZLN mice exhibited significantly more dendritic spines and a greater number of dendritic intersections than the SurVeh mice (Figure 7H-K), indicating a restoration of dendritic complexity and synaptic structures. Behaviorally, all groups had similar performance during training phase (Figure 7L, M). In the probe trial on postoperative day 3, the SurZLN mice crossed the former platform location more frequent and spent longer time in the target quadrant than the SurVeh mice (Figure 7N-P). There was no difference in swimming speed among the Veh, SurVeh and SurZLN groups (Figure 7Q). In the Y-maze spontaneous alternation test, the SurZLN group exhibited a significantly higher alternation rate than the SurVeh group (Figure 7R-S), indicating an improved spatial working memory.

### Microglial PGC-1α overexpression alleviated synaptic pruning and improved hippocampal synaptic plasticity and cognitive function following anesthesia and surgery in aged mice

**Figure 8:**
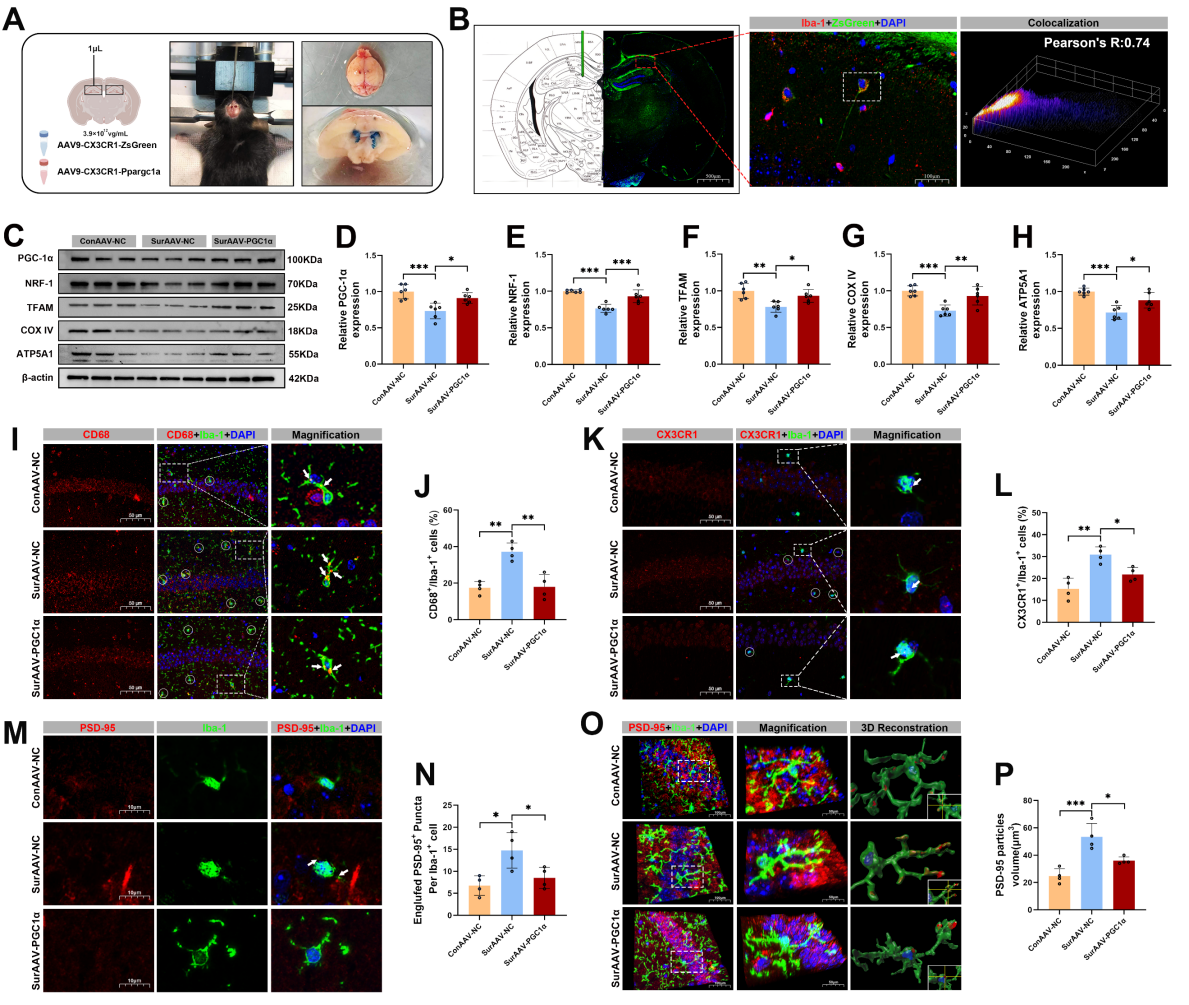
** Effects of AAV-mediated PGC-1α overexpression in microglia on mitochondrial function and hippocampal synaptic pruning following anesthesia and surgery in aged mice.** (A) Schematic diagram of *in vivo* hippocampal injection of adeno-associated virus; (B) Representative Immunofluorescence co-localization images of ZsGreen and Iba-1; (C) Representative Western blots of PGC-1α, NRF-1, TFAM, COX IV and ATP5A1 in the hippocampus; (D-H) The relative expression levels of PGC-1α, NRF-1, TFAM, COX IV and ATP5A1 in the hippocampus, n=6; (I) Representative double-immunofluorescence images of CD68 (red) and Iba-1 (green) in the hippocampal CA1 region; (J) Quantificative analysis for CD68^+^/Iba-1^+^ cells in the hippocampal CA1 region, n=4; (K) Representative double-immunofluorescence images of CX3CR1 (red) and Iba-1 (green) in the hippocampal CA1 region; (L) Quantificative analysis for CX3CR1^+^/Iba-1^+^ cells in the hippocampal CA1 region, n=4; (M) Representative double-immunofluorescence images of PSD-95 (red) and Iba-1 (green) in the hippocampal CA1 region; (N) Quantificative analysis for engulfed PSD-95^+^ puncta co-located with Iba-1 per cells in the hippocampal CA1 region, n=4; (O) Representative double-immunofluorescence with 3D surface reconstruction images of PSD-95 (red) and Iba-1 (green) in the hippocampal CA1 region; (P) Quantificative analysis for PSD-95 particles volume in Iba-1^+^ cells in the hippocampal CA1 region, n=4. Data are presented as the mean ± SD. One-way ANOVA followed by Bonferroni's multiple comparisons test was used to obtain *p*-value. *p <0.05, **p < 0.01, ***p < 0.001.

To clarify the specific role of PGC-1α in hippocampal microglia after anesthesia and surgery in aged mice, we selectively overexpressed microglial PGC-1α by hippocampal microinjection of AAV9-CX3CR1-Ppargc1a (Figure 8A). Immunostaining confirmed that Iba-1⁺ microglia in the hippocampus were strongly co-localized with ZsGreen, indicating efficient and microglia-specific viral infection (Figure 8B). Consistently, Western blot analysis showed a significantly increased expression of hippocampal PGC-1α in the Sur-AAV-PGC1α group compared with the Sur-AAV-NC group (Figure 8C, D). Moreover, PGC-1α overexpression was accompanied by upregulations of mitochondrial biogenesis-related proteins NRF-1, TFAM, COX IV, and ATP5A1 in the Sur-AAV-PGC1α group (Figure 8C, E-H). To further assess microglial phagocytosis of synapses, we co-stained CD68 and CX3CR1 in hippocampal tissues from post-surgery mice. As shown in Figure 8I-L, microglial PGC-1α overexpression significantly reduced the proportion of CD68⁺/CX3CR1⁺ microglia, suggesting an inhibitory effect of PGC-1α on microglial phagocytic activation. Consistently, the co-localization of PSD-95 within the Iba-1⁺ microglia was significantly decreased in the Sur-AAV-PGC1α group relative to the Sur-AAV-NC group (Figure 8M-P).

**Figure 9:**
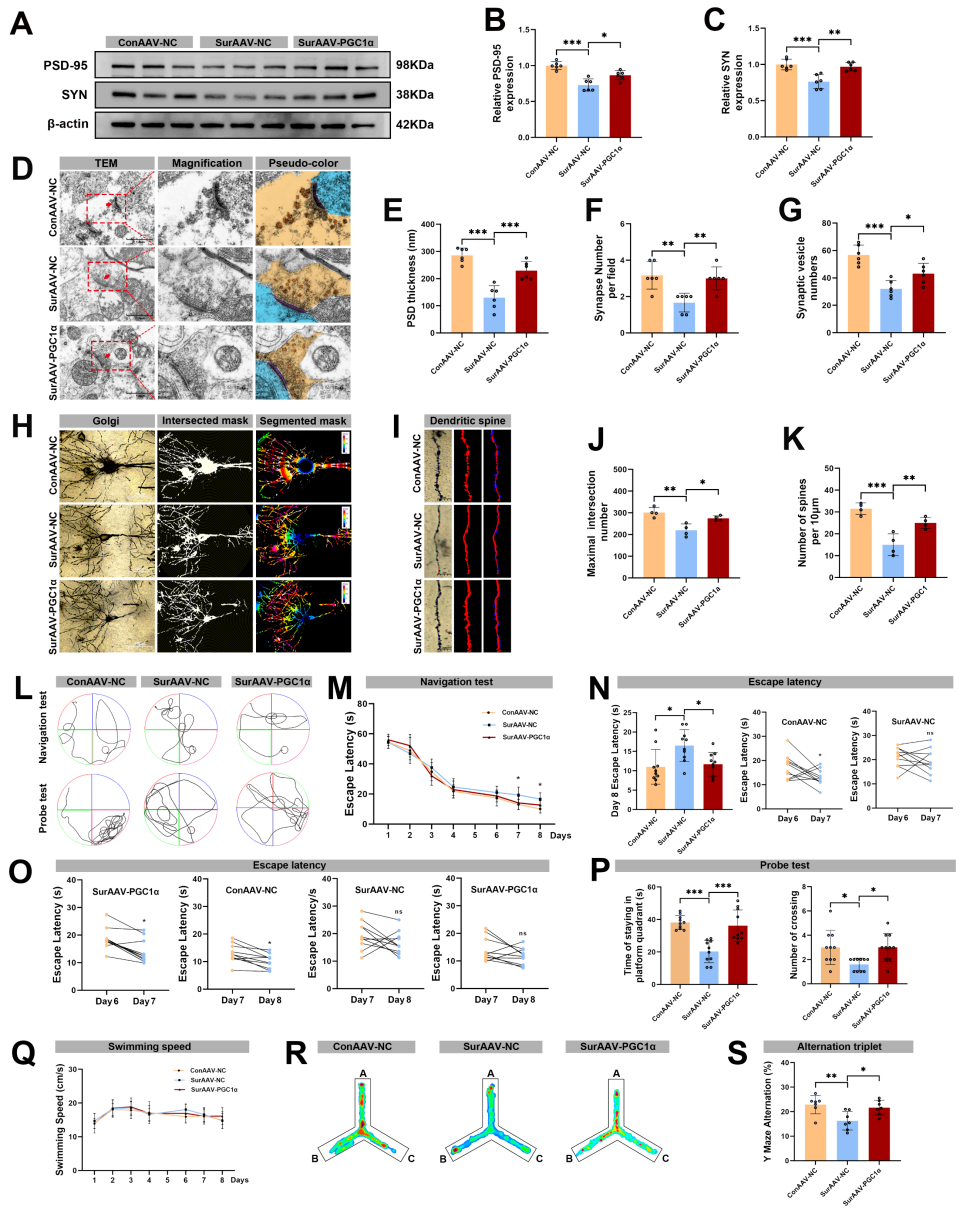
** Effects of AAV-mediated PGC-1α overexpression in microglia on synaptic plasticity and cognitive function following anesthesia and surgery in aged mice.** (A) Representative Western blots of PSD-95 and SYN in the hippocampus of aged mice; (B) The relative expression levels of PSD-95 and SYN in the hippocampus, n=6; (D) Representative TEM images of synapse in the hippocampal CA1 region; (E-G) Quantificative analysis for the thickness of postsynaptic density, synaptic vesicles, and synapse number in the hippocampal CA1 region by TEM, n=6; (H) Representative Golgi-cox staining images of neurons in the hippocampal CA1 region; (I) Representative Golgi-cox staining with Sholl analysis images of neurons in the hippocampal CA1 region; (J) Quantificative analysis for maximum number of intersection points in the hippocampal CA1 region, n=4; (K) Quantificative analysis for the number of dendritic spines, n=4; (L) Trajectories of swimming in the MWM test; (M-O) Escape latency in the MWM test, n=10; (P) Time of staying on the platform and number of crossing the platform, n=10; (Q) Swimming speed in the MWM test, n=10; (R) Heatmap of paths in the Y-maze test; (S) Y-maze spontaneous alternation rate, n=7. Data are presented as the mean ± SD. One-way ANOVA followed by Bonferroni's multiple comparisons test was used to obtain *p*-value. *p <0.05, **p < 0.01, ***p < 0.001, ns, not significant.

In accordance with these observations, expression levels of synaptic markers PSD-95 and SYN in hippocampus were significantly higher in the Sur-AAV-PGC1α group than in the Sur-AAV-NC group (Figure 9A-C). The TEM further demonstrated that PGC-1α overexpression increased PSD thickness, synaptic vesicle number, and total synapse number in the hippocampal neurons (Figure 9D-G). Additionally, the Sholl analysis revealed a significant increase in the maximum number of dendritic intersections, accompanied by a higher dendritic spine density, in the Sur-AAV-PGC1α group compared with Sur-AAV-NC group (Figure 9H-K). Finally, behavioral tests were conducted to assess whether microglial PGC-1α overexpression could ameliorate the cognitive deficits induced by anesthesia and surgery. During the training phase, all groups performed comparably. However, the Sur-AAV-PGC1α group exhibited a significantly shorter mean escape latency post-surgery compared with the Sur-AAV-NC group (Figure 9L-O). In the probe trial, the Sur-AAV-PGC1α mice spent more time in the target quadrant and crossed the former platform location more frequently than controls (Figure 9P). There was no difference in swimming speed among the ConAAV-NC, SurAAV-NC and SurAAV-PGC1α groups (Figure 9Q). Additionally, in the Y-maze test, spontaneous alternation rates were significantly higher in the Sur-AAV-PGC1α group compared with the Sur-AAV-NC group (Figure 9R-S). Altogether, these results indicate that overexpression of microglial PGC-1α in the hippocampus of aged mice suppresses excessive synaptic phagocytosis and synaptic loss, preserves synaptic plasticity, and improves cognitive outcomes. These results suggest a promising strategy for mitigating POCD.

## Discussion

POCD is an increasingly neurological complication following surgery, especially in elderly patients undergoing major operations. Although the 2018 international consensus recommended the use of the umbrella term “perioperative neurocognitive disorders (PND)” in the clinical studies and practices 51, we retained “POCD” in this experiment to maintain consistency with the large body of preclinical and animal-model literatures that continue to use this term. In this experiment, we examined the changes of PGC-1α expression in the hippocampal microglia of aged mice after anesthesia and surgery, and explored its impacts on synaptic plasticity and cognition. The results showed that post-surgery, PGC-1α expression in the hippocampal microglia was significantly reduced, accompanied by disrupted mitochondrial energy metabolism. Loss of PGC-1α triggered aberrant microglial phagocytosis and excessive synaptic pruning, leading to synaptic loss and POCD. Crucially, either pharmacological activation of PGC-1α or microglia-specific overexpression of PGC-1α restored mitochondrial energy metabolism, suppressed microglial synaptic engulfment, preserved synaptic integrity, and improved postoperative cognitive outcomes. These findings indicate that microglial PGC-1α could serve as an effective therapeutic target to safeguard synaptic plasticity and cognitive function in the postoperative period.

PGC-1α is widely regarded as a master regulator of mitochondrial homeostasis, oxidative phosphorylation (OXPHOS) and inflammatory response, thereby preserving mitochondrial quality, sustaining energy production, and maintaining redox balance 29. In the central nervous system (CNS), mounting evidence supports PGC-1α's neuroprotective capacity, both in neurons and glial cells. Early work demonstrates that PGC-1α overexpression in the hippocampal neurons enhances dendritic spine formation and synaptogenesis, while PGC-1α knockdown reduces spine density *in vivo*, indicating the roles of neuronal PGC-1α in spinogenesis and synaptic maintenance 52. Similarly, in the neurodegenerative or ischemic models, PGC-1α overexpression in the glial cells (including microglia) has been reported to enhance mitophagy, reduce reactive oxygen species, suppress inflammasome activation, and attenuate neuroinflammation 31, 53. These findings imply that mitochondrial health in the glial cells may directly influence synaptic maintenance and neuroprotection. Our study extends these observations by showing that, in microglia, PGC-1α is not merely important for inflammatory regulation or oxidative stress, but also critically influences microglial phagocytic behavior and synaptic pruning. In our POCD model, the reduction of hippocampal PGC-1α is accompanied by downregulation of mitochondrial biogenesis factors (e.g. NRF-1, TFAM), diminished expression of OXPHOS proteins (COX IV, ATP5A1), and decreased ATP content. These mitochondrial perturbations correlated with morphological microglial activation, upregulation of phagocytic markers (CD68, CX3CR1), increased engulfment of synaptic components (PSD-95), reduction of synaptic protein levels and dendritic spine density, deterioration of synaptic ultrastructure, and impaired cognitive behavior.

Despite extensive evidence supporting microglial heterogeneity, the classical M1/M2 polarization does not sufficiently capture the full spectrum of activation states, and the definitive criteria for microglial subtypes specifically involved in synaptic pruning remain elusive 54, 55. RNA sequencing has emerged as a powerful approach for resolving such transcriptionally defined microglial clusters with high resolution 56. Our snRNA-seq analysis identified seven distinct microglial subtypes in the hippocampus post-surgery. Among these, the HOM and IAM clusters were enriched for the transcripts associated with phagocytic function (e.g., P2ry12, CD68) and chemokine signaling (e.g., CX3CR1), suggesting their prominence in the post-surgical synaptic remodeling. Notably, although snRNA-seq did not detect the robust increases in CD68 or CX3CR1 transcripts across the entire microglial population after surgery, the the hippocampal CD68 and CX3CR1 protein levels were significantly elevated. This mismatch between the transcripts and protein changes likely reflects the post-transcriptional regulatory mechanisms and differences in the translation or subcellular trafficking that are not fully captured by snRNA-seq, as this nucleus-centric approach predominantly profiles nuclear transcripts and may underrepresent cytoplasmic or membrane-associated RNA species and their encoded proteins 57, 58. Importantly, microglial PGC-1α expression was downregulated after anesthesia and surgery, supporting its relevance as a molecular indicator of microglia involved in synaptic pruning. The complementary CellChat analysis indicated the strengthened inferred interactions between microglia and both inhibitory and excitatory neurons in the hippocampus post-surgery, further implicating altered microglial signaling in the pathogenesis of POCD.

The pre-surgical treatment of ZLN005, a small-molecule activator of PGC-1α, showed that pharmacological activation of PGC-1α could prevent the POCD associated pathophysiological changes. That is, ZLN005 treatment enhanced PGC-1α transcriptional activity and strengthened its interaction with transcription factors such as NRF-1 and TFAM, thereby stimulating mitochondrial biogenesis and maintaining mitochondrial energy metabolism 59-61. Other than its effects on mitochondria, ZLN005 has also been demonstrated significant antioxidant and anti-inflammatory properties that may act independently of mitochondrial biogenesis 62, 63. Given these converging lines of evidence, while our data strongly implicate the role of PGC-1α pathway in the beneficial effects of ZLN005, the results of our rescue experiments should be interpreted with appropriate caution. The protective effects of ZLN005 likely represent the results from a combination of different mechanisms, including restoration of mitochondrial function, enhanced oxidative metabolism, and anti-inflammatory activity, and may occur across multiple cell types rather than being strictly microglia-specific. To further clarify this issue, an AAV9 vector encoding PGC-1α under the control of a (putatively) microglia-specific CX3CR1 promoter was used to drive PGC-1α overexpression in the hippocampal microglia, following the established protocols in the published studies including our prior work 41-43. Remarkably, overexpression of microglial PGC-1α alone was sufficient to restore mitochondrial function, reduce excessive synaptic pruning, preserve synaptic integrity, and improve cognitive performance. Therefore, these results provide compelling evidence that microglial PGC-1α represents a critical regulator linking mitochondrial metabolism, microglial phagocytic behavior, synaptic remodeling, and cognitive outcomes in the postoperative context.

An increasing number of studies have linked microglia-mediated synaptic pruning and synaptic loss to the development of POCD 11, 64, yet the precise mechanisms underlying surgery-induced aberrant microglial synapse pruning remain incompletely understood. Microglial energy metabolism has been reported to play a central role in regulating their activation and phagocytic behavior. Under physiological conditions, microglia depend largely on mitochondrial OXPHOS to generate ATP and support surveillance, motility, and synaptic maintenance 65. In contrast, inflammatory stimuli such as LPS can shift microglial metabolism from OXPHOS toward glycolysis, causing reduced oxygen consumption, increased lactate production, and impaired mitochondrial respiration 66. This metabolic shift supports a reactive, pro-inflammatory microglial phenotype, with elevated expression of phagocytic markers and heightened phagocytic activity 67. It has been demonstrated that dysfunction of homeostatic microglia, for example microglia activated by LPS, can lead to synapse loss in primary neuronal cultures, likely due to synaptotoxic factors secreted by microglia 68. In our neuron-microglia co-culture system, we observed that microglia with compromised mitochondrial metabolism display not only enhanced activation but also increased synaptic phagocytic activity, thereby establishing a functional link between metabolic impairment and aberrant synapse removal under perioperative conditions. Crucially, we provided novel mechanistic insight by showing that activation of PGC-1α in BV-2 microglial cells with ZLN005 restored mitochondrial energy metabolism, and significantly reduced PSD-95 phagocytosis. These findings indicate that microglial synaptic phagocytosis is tightly regulated by mitochondrial energy metabolism, and that excessive synaptic pruning leads to enhanced pathological microglia-neuron interactions following anesthesia and inflammatory challenges.

Upregulation of CD68 in the microglia is indicative of enhanced phagocytic activity and has been associated with increased microglial engulfment of synapses in diverse neuropathological setting 69. Beyond phagolysosomal markers, the CX3CR1-CX3CL1 axis plays a pivotal role in microglia-neuron communication, that is, neuron-derived CX3CL1 restrains microglial activation and limits synaptic engulfment, whereas CX3CR1, expressed predominantly on microglia, mediates eat-me signal and contributes to synapse elimination 70. Emerging evidence indicates that anesthesia and surgical stress can disrupt this signal axis, with reduced neuronal CX3CL1 and concomitantly increased microglial CX3CR1, weakening neuron-derived inhibitory cues and biasing microglia toward a reactive, phagocytic state in postoperative models 64, 71. Extending these findings, our experiment showed that anesthesia and surgical stress not only elevated CX3CR1 expression, but also increases CD68 expression in hippocampal microglia, indicating a shift toward a more phagocytic and reactive phenotype. Importantly, the restoration of microglial PGC-1α reestablished mitochondrial homeostasis, attenuated upregulation of both CD68 and CX3CR1, and reduced aberrant synaptic pruning, thereby preserving synaptic integrity. These results suggest that impaired fractalkine signaling and intrinsic metabolic dysfunction synergize to drive excessive microglial engulfment in POCD, and identify PGC-1α as a key upstream regulator of microglial structural remodeling, highlighting a novel metabolic contribution to dysregulated microglial responses. This experiment has several limitations. First, the pharmacological activator ZLN005 is not strictly microglia-specific, and its beneficial effects may involve other cell types or non-PGC-1α-dependent actions, such as antioxidant or anti-inflammatory effects. While we used microglia-targeted AAV to mitigate this concern, absolute cell-type specificity and potential off-target effects cannot be excluded. Transgenic mice with microglia-specific PGC-1α overexpression would provide a more definitive conclusion in future studies. Second, we focused on the clinically relevant “double-hit” model of anesthesia plus surgical trauma and did not include an anesthesia-alone group. This limits the ability to fully disentangle the effects of anesthesia from those of surgical trauma. Third, while the Morris Water Maze and Y-maze have been widely validated in POCD researches and sensitive to hippocampal-dependent deficits in non-aversive contexts, the absence of an associative memory test (e.g., fear conditioning) is a limitation. Including such tests could provide valuable insight into emotional or contextual memory domains. Finally, we investigated the role of microglial PGC-1α in a POCD animal model, but human data on PGC-1α expression in POCD patients is needed to strengthen its translational relevance. Future longitudinal clinical studies, such as serial sampling of peripheral blood mononuclear cells (PBMCs) or cerebrospinal fluid (CSF) from elderly surgical patients, are essential to assess PGC-1α and related mitochondrial markers. This is crucial before PGC-1α can be proposed as a biomarker or therapeutic target for POCD.

In conclusion, this experiment uncovers a novel molecular mechanism in which microglial PGC-1α serves as a key mediator in the pathogenesis of POCD, connecting mitochondrial energy metabolism to microglia-mediated synaptic pruning. These findings highlight microglial PGC-1α as a promising therapeutic target for the prevention and treatment of POCD.

## Supplementary Material

Supplementary figures.

## Data Availability

All data supporting the findings of this study are available from the corresponding author upon reasonable request.
