# Supplementary material for: Microglial PGC-1α alleviates synaptic damage and cognitive impairments following anesthesia and surgery by suppressing excessive synaptic pruning in aged mice
Source: Int J Biol Sci. 2026 Mar 25;22(7):3635–57. doi: 10.7150/ijbs.121472 (PMC13086008; doi:10.7150/ijbs.121472)
Supplement: Supplementary file 1 — Supplementary figures. [file ijbsv22p3635s1.pdf]

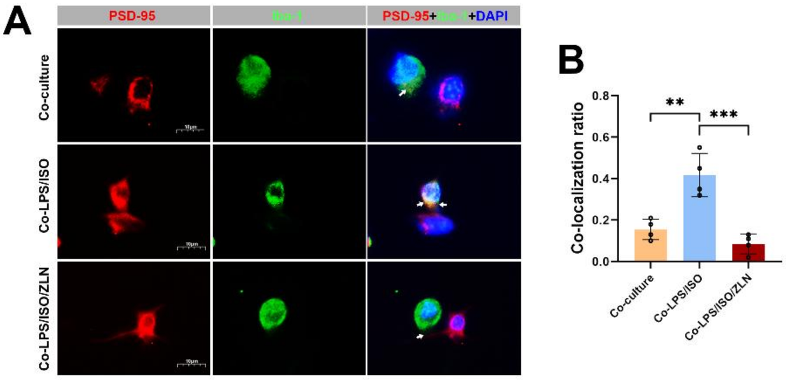

Figure S1 Activation of PGC-1α reduces synaptic phagocytosis in vitro

(A) Representative double-immunofluorescence images of PSD-95 (red) and Iba-1 (green) in co-cultured cells, n=4; (B) Quantification of co-localization ratio of PSD-95 and Iba-1, n=4. Data are presented as the mean ± SD. One-way ANOVA followed by Bonferroni's multiple comparisons test was used to obtain *p*-value. $^{**}p < 0.01$, $^{***}p < 0.001$.

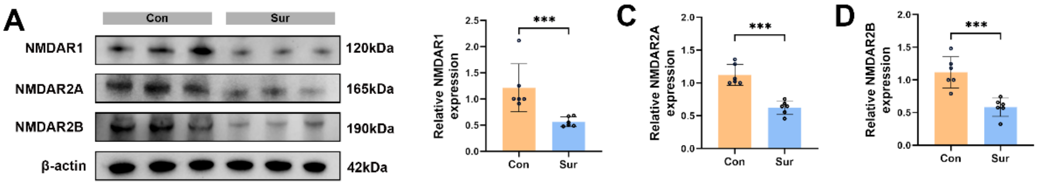

Figure S2 Postsynaptic protein expression reduced following anesthesia and surgery in aged mice

(A) Representative Western blots of NMDAR1, NMDAR2A and NMDAR2B in the hippocampus; (B-D) The relative expression levels of NMDAR1, NMDAR2A and NMDAR2B in the hippocampus, n=6; One-way ANOVA followed by Bonferroni's multiple comparisons test was used to obtain *p*-value. $^{***}p < 0.001$.
